# Version 6 of the consensus yeast metabolic network refines biochemical coverage and improves model performance

**DOI:** 10.1093/database/bat059

**Published:** 2013-08-09

**Authors:** Benjamin D. Heavner, Kieran Smallbone, Nathan D. Price, Larry P. Walker

**Affiliations:** ^1^Institute for Systems Biology, 401 Terry Ave N., Seattle, WA 98109, USA, ^2^Manchester Centre for Integrative Systems Biology, University of Manchester, Manchester, M1 7DN, UK and ^3^Biomass Conversion Laboratory, Department of Biological and Environmental Engineering, Cornell University, Ithaca, NY 14853, USA

## Abstract

Updates to maintain a state-of-the art reconstruction of the yeast metabolic network are essential to reflect our understanding of yeast metabolism and functional organization, to eliminate any inaccuracies identified in earlier iterations, to improve predictive accuracy and to continue to expand into novel subsystems to extend the comprehensiveness of the model. Here, we present version 6 of the consensus yeast metabolic network (Yeast 6) as an update to the community effort to computationally reconstruct the genome-scale metabolic network of *Saccharomyces cerevisiae* S288c. Yeast 6 comprises 1458 metabolites participating in 1888 reactions, which are annotated with 900 yeast genes encoding the catalyzing enzymes. Compared with Yeast 5, Yeast 6 demonstrates improved sensitivity, specificity and positive and negative predictive values for predicting gene essentiality in glucose-limited aerobic conditions when analyzed with flux balance analysis. Additionally, Yeast 6 improves the accuracy of predicting the likelihood that a mutation will cause auxotrophy. The network reconstruction is available as a Systems Biology Markup Language (SBML) file enriched with Minimium Information Requested in the Annotation of Biochemical Models (MIRIAM)-compliant annotations. Small- and macromolecules in the network are referenced to authoritative databases such as Uniprot or ChEBI. Molecules and reactions are also annotated with appropriate publications that contain supporting evidence. Yeast 6 is freely available at http://yeast.sf.net/ as three separate SBML files: a model using the SBML level 3 Flux Balance Constraint package, a model compatible with the MATLAB® COBRA Toolbox for backward compatibility and a reconstruction containing only reactions for which there is experimental evidence (without the non-biological reactions necessary for simulating growth).

**Database URL:** http://yeast.sf.net/

## Introduction

In 2007, a community effort to integrate previously published genome-scale reconstructions of the yeast metabolic network (1, 2) produced a ‘consensus’ representation of yeast metabolism (3), which has subsequently been updated through iterative collaborative curation by multiple research groups (4, 5). Here, we introduce version 6 of the consensus reconstruction of the yeast metabolic network, Yeast 6. The differences between Yeast 5 and Yeast 6 are described below and are fully detailed in the supplementary data attached to this publication. This update maintains an emphasis on standards compliance, unambiguous metabolite naming and computer-readable annotations available through a structured document format. Additionally, we have developed MATLAB® scripts to demonstrate our approach for comparing Yeast 5 and Yeast 6 using flux balance analysis (FBA) methods, leveraging on the COnstraint-Based Reconstruction and Analysis (COBRA) Toolbox (6). These scripts are also included as supplementary data.

To emphasize the distinction between the established biochemistry included in a metabolic genome-scale network reconstruction (GENRE) (7) and the additional modeling assumptions required for analysis or simulation with a genome-scale model (GEM) (7), we have made Yeast 6 available at http://yeast.sf.net/ as three separate Systems Biology Markup Language (SBML) files: a GEM using the SBML level 3 Flux Balance Constraint package, a GEM compatible with the MATLAB® COBRA toolbox (6) for backward compatibility and a GENRE containing only reactions for which there is experimental evidence.

## Results

### Overview and network characteristics

Yeast 6 resulted from an effort to improve the predictive accuracy of Yeast 5 through manual curation, with particular focus on removing information that is not well supported by published literature and by adding metabolic pathway information that has been recently discovered. As a result of this effort, Yeast 6 contains fewer metabolites and reactions than Yeast 5 (Table 1), but is more accurate in its predictions of gene essentiality (Table 2) and auxotroph-inducing mutations (Table 3). Ninety-seven of the 1868 reactions shared between Yeast 5 and Yeast 6 have different constraints, reflecting refinements of reaction reversibility in the yeast metabolic network.

**Table 1.** Summary statistics of Yeast 5 and Yeast 6

Summary statistics	Yeast 5	Yeast 6
Genes	918	900
Metabolites	1655	1458
Reactions	2110	1888
Reactions with PMID references	37.6%	40.4%

Because of additional quality curation, Yeast 6 has fewer blocked reactions and a greater percentage of reactions annotated with literature evidence; it also contains fewer genes, metabolites and reactions than Yeast 5. Neither reconstruction includes open reading frames annotated as ‘dubious’ in the Saccharomyces Genome Database (7).

**Table 2.** Comparing gene essentiality predictions of Yeast 5 and Yeast 6

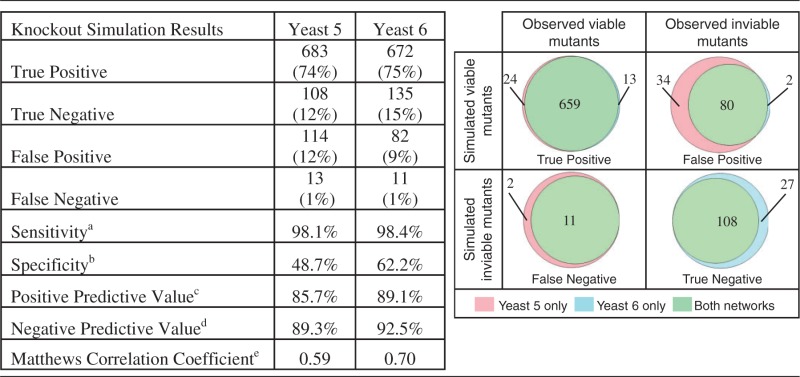

^a^TP/(TP + FN)

^b^TN/(TN + FP)

^c^TP/(TP + FP)

^d^TN/(TN + FN)

^e^
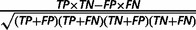
(8)

Yeast 6 has fewer false-positive predictions and more true-negative predictions of gene essentiality, leading to improvements in sensitivity, specificity, positive predictive value, negative predictive value and Matthews correlation coefficient (see Discussion for more information on the use of this metric).

**Table 3.** Comparing auxotroph mutant predictions of Yeast 5 and Yeast 6

Simulation Results	Yeast 5	Yeast 6
Auxotroph-inducing genes	92	93
Correct auxotrophy predictions	57	64
Mutant incorrectly predicted to be viable in minimal medium	32	26
Mutant incorrectly predicted to be inviable in supplemented medium	3	3

Yeast 6 has more genes for which a deletion has been reported to cause auxotrophy and has more correct predictions of such auxotrophy than Yeast 5.

### Novel features of Yeast 6

Metabolites, reactions and genes differ between Yeast 5 and Yeast 6. Detailed lists of common and distinct metabolites, reactions, genes, constraints, auxotrophs and knockout predictions are included as supplementary data, as is the code used to generate these comparisons. The supplementary data also include specific rationale for reaction deletions and additions, as well as detailed descriptions of the function of each gene removed or added. In summary, Yeast 5 has 203 metabolites that are not in Yeast 6, whereas Yeast 6 introduces 6 new metabolites. The smaller number of metabolites in Yeast 6 arises from the removal of metabolites involved in those dead-end reactions annotated as a modeling reaction (i.e. it was included in Yeast 5 without biochemical or genomic evidence). No dead-end reactions that are annotated with a gene were removed. Yeast 5 has 242 reactions that are not in Yeast 6, whereas Yeast 6 has 20 reactions that are not in Yeast 5. As detailed in supplementary data, reactions removed in Yeast 6 include those with incorrect cofactor specificity, those involving protein modification, those not supported upon literature review and dead-end modeling reactions. Yeast 5 has 31 genes that are not in Yeast 6, and Yeast 6 has 13 genes that are not in Yeast 5. Of the 31 genes removed, 21 encode proteins involved in protein modification or Glycosylphosphatidylinositol (GPI)-anchor assembly (processes that are not strictly metabolic), 4 are annotated with ‘putative’ function, 1 was removed because of unclear cofactor specificity, 2 annotated poorly characterized reactions involved with ergosterol biosynthesis, 1 encodes a flippase, which would require a finer level of membrane compartmentalization than currently exists in the model, and the metabolic function of the remaining 2 are not well established.

### Essentiality and auxotrophy predictions with Yeast 6

These changes to the reconstructed metabolic network, combined with the incorporation of suggested changes to the biomass definition (8), give rise to altered FBA predictions of gene essentiality in glucose-limited aerobic conditions and to altered predictions of auxotrophy induced through gene deletion. When compared with a list of essential genes we compiled (this list is included in the supplementary data *testYeast.m* file), Yeast 6 has 13 new true positive predictions of gene essentiality (i.e. FBA predicts that biomass can be produced following the deletion of inessential genes), 27 new true negative predictions (i.e. FBA predicts that biomass cannot be produced when essential genes are deleted), 1 new gene predicted to induce auxotrophy following mutation and 7 newly correct predictions of auxotrophy phenotypes. Yeast 6 also introduces new predictions that differ from laboratory observations, two new false-positives (growth predicted despite the deletion of an essential gene), one new auxotrophic mutant predicted incorrectly to be incapable of growth in supplemented media and two new mutants incorrectly predicted to be viable in minimal media despite reported auxtrophy. We note that such differences between model prediction and observation may arise from regulatory constraints that are outside the scope of a metabolic reconstruction, or may be informative of opportunities for continued network curation.

### Yeast 6 aerobic and anaerobic growth

Unlike Yeast 5, Yeast 6 does not include separate biomass definitions for simulating aerobic and anaerobic growth. Yeast 6 correctly predicts that yeast will not grow on minimal media in strict anaerobic conditions [*Saccharomyces cerevisiae* requires supplementation with unsaturated fatty acids and sterols (9, 10)]. Simulating anaerobic growth with Yeast 6 requires the simulated medium to be supplemented with sterols [i.e. the exchange reactions for episterol, ergosterol, fecosterol, lanosterol, zymosterol and ergosta-5,7,22,24(28)-tetraen-3beta-ol must have nonzero lower bounds].

### Data and annotation standards

The network reconstruction is provided as an SBML (11) file enriched with MIRIAM-compliant (12) annotations. Small- and macromolecules are referenced to community-standard databases such as Uniprot (13) or ChEBI (14). Molecules and reactions are also annotated with appropriate publications that contain supporting evidence. Thus, this network is presented in a computational framework that adheres to community standards and is entirely traceable. To facilitate comparison between reconstructions and models, Yeast 6 metabolite and reaction identifiers are consistent with Yeast 5 identifiers (e.g. reaction ‘r_0123’ in Yeast 5 is the same reaction as ‘r_0123’ in Yeast 6, and metabolite ‘s_0042’ in Yeast 5 is the same metabolite as ‘s_0042’ in Yeast 6).

Yeast 6 follows the same modeling conventions as Yeast 5. We used the SBML specification for encoding reaction and metabolite annotation rather than the COBRA Toolbox-specific convention of using a custom ‘Notes’ field. Our sign convention for exchange reactions is that positive flux values represent compounds produced in FBA simulation, and negative flux values represent compounds consumed, and we include biomass as a specific species in the model.

### Assessing metabolic models

Because a gene picked at random is more likely to be inessential than essential for growth (15), overall accuracy is not a good metric for assessing model predictive ability (16). This statistical issue has previously been recognized, leading to the use of the ‘geometric mean accuracy’ as a metric for evaluating metabolic network models (2). Because geometric mean ignores the positive predictive value (also called precision) (17), we report all values of the contingency matrix (Table 2), and summarize the predictive ability of the model with the Matthews correlation coefficient (18), a metric that is robust across a range of prevalence values and incorporates positive and negative predictive values.

We include the testYeast.m script as supplementary data to facilitate evaluation of this model. This script compares model phenotype predictions against lists of verified yeast open reading frames, genes that we consider essential and genes that cause auxotrophy upon deletion. We compiled these lists from the Yeast Deletion Project (15) and from information in the Saccharomyces Genome Database (19).

## Discussion

Yeast 6 is the current state-of-the-art reconstruction of the *S. cerevisiae* metabolic network. It eliminates many inferred reactions for which there is no evidence, adds new reactions based on recent evidence and results in improved predictions of experimental data. It maintains the distinction between GENRE and GEM, and by emphasizing traceable annotation for included information, it differentiates between established biochemistry and hypotheses that may be generated by automated techniques such as gap-filling algorithms (20). It will thus be a useful addition to the consensus resource and to the large community of researchers who use the yeast metabolic model to guide experimental and modeling efforts.

### Limitations

Improving the reconstruction of the yeast metabolic network remains an ongoing project. In addition to the model predictions that differ from experimental observations described above (i.e. false-positive, false-negative and incorrect auxotroph predictions), there remains substantial opportunity to improve the reconstruction of lipid metabolism. This point is most evident from the fact that unsaturated fatty acids are not currently required for simulating anaerobic growth, but also arises through the continued use of generic lipid species [i.e. compounds using generic residual (-R) groups, rather than precise stoichiometrically balanced definitions of fatty acid moieties].

Additional limitations arise from the appropriately limited scope of the metabolic network reconstruction. Condition-dependent constraints that arise from various regulatory mechanisms are not included in Yeast 6. Thus, pathways that are affected through transcriptional regulatory events such as glucose repression may be incorrectly predicted to carry fluxes under FBA (e.g. in the absence of additional constraints, malate can cycle between mitochondrial malate dehydrogenase and cytoplasmic malate dehydrogenase). Integration of regulatory and metabolic networks remains an area of active research (21, 22).

Like other metabolic network models, not all reactions in Yeast 6 can carry flux in FBA simulation. Yeast 6 has 738 blocked reactions (39%), a similar portion of blocked reactions as Yeast 5 (38%). Blocked reactions indicate knowledge limitations (such as reactions leading to dead-end metabolites whose metabolic fate or origin is unknown, or reactions involved in unconnected portions of metabolism, which form unconnected subgraphs in the network). Like FBA predictions that differ from observation, the number of blocked reactions is also affected by condition-specific constraints, particularly constraints on reaction reversibility. We have observed that relaxing the reversibility of reactions involving nucleotide cofactors reduces the number of blocked reactions.

Unlike Yeast 5, the prediction of anaerobic ethanol production in FBA simulation requires manual restriction of the reaction catalyzed by ATP synthase. The requirement for this condition-dependent constraint may arise from the lack of regulatory constraints as described above, or from a need for physicochemical capacity constraints on allowable flux. Additionally, it may reflect other, presently uncharacterized limitations. We note that as with integrating regulatory constraints, detailed reconstruction of cofactor and proton balancing also remains an area of active research in the constraint-based modeling community (23); redox conditions are clearly of critical importance to the function of ATP synthase.

### An invitation to participate in the community effort to reconstruct the yeast metabolic network

Computational reconstruction and modeling of yeast metabolism is an ongoing project, and we invite additional community participation in this effort. Suggestions for improving the yeast consensus reconstruction or derived models should be submitted to network.reconstruction@manchester.ac.uk. Metabolites and enzymes should be unambiguously identified, using existing model or database (ChEBI or UniProt) identifiers. New reactions should be supplied with primary evidence for their mechanism and catalysis, via PubMed identifiers. Reactions without evidence should have clear reasons for their proposed addition.

## Supplementary data

Supplementary data are available at *Database* Online.

Supplementary Data
