# Supplementary material for: Version 6 of the consensus yeast metabolic network refines biochemical coverage and improves model performance
Source: Database (Oxford). 2013 Aug 9;2013:bat059. doi: 10.1093/database/bat059 (PMC3739857; doi:10.1093/database/bat059)
Supplement: Supplementary Data [file supp_2013_bat059_index.html]

Version 6 of the consensus yeast metabolic network refines biochemical coverage and improves model performance — Supplementary Data 

# Version 6 of the consensus yeast metabolic network refines biochemical coverage and improves model performance

## Supplementary Data

files

**Files in this Data Supplement:**

- Supplementary Data - pdf file
- Supplementary Data - pdf file
- Supplementary Data - m file
- Supplementary Data - xlsx file
- Supplementary Data - m file
