# Supplementary material for: Version 6 of the consensus yeast metabolic network refines biochemical coverage and improves model performance
Source: Database (Oxford). 2013 Aug 9;2013:bat059. doi: 10.1093/database/bat059 (PMC3739857; doi:10.1093/database/bat059)
Supplement: Supplementary Data [file supp_bat059_compareModelseg.pdf]

```
function [per_model comparison]=compareModels(varargin)

% compareModels: a tool for comparing versions of the Consensus
% Reconstruction. For now, good for comparing 2 models.
%
% Tested with:
%             Yeast 5 - doi:10.1186/1752-0509-6-55
%             Yeast 6 - http://yeast.sf.net (to be published)
%
% Input:
%   models    A structure containing COBRA Toolbox-format yeast models
%             (e.g. models(1)=Y5; models(2)=Y6;). This code currently
%             compares the first 2 models.
%
%   results (optional)
%             If a structure of results generated from testYeast.m is
%             provided, compareModels will run the comparison on this
%             structure. If not, compareModels will call testYeast using
%             the models in the models structure.
%
% Output:
%   per_model   a structure containing the following fields for each model:
%             description = model.description
%             medium = medium used
%             genes = number of genes in model
%             dubious = number of dubious ORFs in model
%             metabolites = number of metabolites in model
%             reactions = number of reactions in model
%             blocked = number of blocked reactions
%             TP = numer of true positive KO predictions (model predicts
%                    growth when nonessential genes are deleted)
%             TN = numer of true negative KO predictions
%             FP = numer of false positive KO predictions
%             FN = numer of false negative KO predictions
%             sensitivity = recall = tp/(tp+fn)
%             specificity = tn/(tn+fp)
%             PPV = positive predictive value = precision = tp/(tp+fp)
%             NPV = negative predictive value = tn/(fn+tn)
%             mean = geometric mean accuracy = (sensitivity + specificity /2)
%             auxotrophs = auxotroph-inducing genes in model
%             auxotroph_correct = number of auxotrophs correctly predicted
%             auxotroph_viable = auxotrophs incorrectly predicted to be
%                    viable in miminal media
%             auxotroph_inviable = auxotrophs incorrectly predicted to be
%                    inviable in supplemented media
%
%   comparison  a structure containing the following lists as cell arrays:
%             common_mets
%             unique_mets_1
%             unique_mets_2
%             common_rxns
%             unique_rxns_1
%             unique_rxns_2
%             common_genes
%             unique_genes_1
%             unique_genes_2
%             common_TP
%             unique_TP_1
%             unique_TP_2
%             common_TN
```

```
%               unique_TN_1
%               unique_TN_2
%               common_FP
%               unique_FP_1
%               unique_FP_2
%               common_FN
%               unique_FN_1
%               unique_FN_2
%               common_auxotrophs
%               unique_auxotrophs_1
%               unique_auxotrophs_2
%               common_auxotrophs_correct
%               unique_auxotrophs_correct_1
%               unique_auxotrophs_correct_2
%               common_auxotrophs_viable
%               unique_auxotrophs_viable_1
%               unique_auxotrophs_viable_2
%               common_auxotrophs_inviable
%               unique_auxotrophs_inviable_1
%               unique_auxotrophs_inviable_2
%               same_lb_constraints - shared reactions with same lb constraints
%               differing_lb_constraints - shared reactions with different lb constraints
%               same_ub_constraints - shared reactions with same ub constraints
%               differing_ub_constraints - shared reactions with different ub constraints


% written BH 7 Feb 2013

%% citation
%
% please cite: Heavner, Benjamin D., Kieran Smallbone, Nathan Price, and
% Larry P. Walker. “Version 6 of the Consensus Yeast Metabolic Network.”
% NEED TO ADD CITATION DETAILS

%% process input arguments
% 2 inputs at most
numvarargs = length(varargin);

if numvarargs > 2
    error('myfuns:comparemodels:TooManyInputs', ...
        'requires at most 2 inputs');
end

models = varargin{1};

if numvarargs == 2;
    results = varargin{2};
end

if length(models) > 2
    fprintf('\ncompareModels will compare the first two models.\n');
end

if length(models) < 2
    error('Need at least 2 models to compare.\n');
end

%% test models

if ~exist('results','var')
```

```
    for index=1:length(models)
        results(index) = testYeast(models(index), 0, 1, 0);
    end
end

%% build per_model

per_model = struct([]);
for index=1:length(results)
    per_model(index).description = results(index).description;
    per_model(index).medium = results(index).medium;
    per_model(index).genes = length(models(index).genes);
    per_model(index).dubious = length(results(index).dubious);
    per_model(index).metabolites = length(models(index).mets);
    per_model(index).reactions = length(models(index).rxns);

    if isfield(results(index),'blocked')
        per_model(index).blocked = length(results(index).blocked);
    end

    per_model(index).TP = length(results(index).TP);
    per_model(index).TN = length(results(index).TN);
    per_model(index).FP = length(results(index).FP);
    per_model(index).FN = length(results(index).FN);

    per_model(index).sensitivity = ...
        per_model(index).TP/(per_model(index).TP + per_model(index).FN);
    per_model(index).sensitivity = per_model(index).sensitivity * 100;
    per_model(index).specificity = ...
        per_model(index).TN/(per_model(index).TN + per_model(index).FP);
    per_model(index).specificity = per_model(index).specificity * 100;

    per_model(index).PPV = ...
        per_model(index).TP/(per_model(index).TP + per_model(index).FP);
    per_model(index).PPV = per_model(index).PPV * 100;
    per_model(index).NPV = ...
        per_model(index).TN/(per_model(index).TN + per_model(index).FN);
    per_model(index).NPV = per_model(index).NPV * 100;
    per_model(index).mean = ...
        (per_model(index).sensitivity + per_model(index).specificity)/2;

    per_model(index).auxotrophs = length(results(index).auxotrophs);
    per_model(index).auxotroph_correct = ...
        length(results(index).auxotrophs) - ...
        length(results(index).auxotroph_viable) - ...
        length(results(index).auxotroph_inviable);
    per_model(index).auxotroph_viable = ...
        length(results(index).auxotroph_viable);
    per_model(index).auxotroph_inviable = ...
        length(results(index).auxotroph_inviable);
end

%% build comparison

comparison = struct('common_mets',{intersect(models.mets)});
comparison.unique_mets_1 = setdiff(models(1).mets,models(2).mets);
comparison.unique_mets_2 = setdiff(models(2).mets,models(1).mets);

comparison.common_rxns = intersect(models.rxns);
comparison.unique_rxns_1 = setdiff(models(1).rxns,models(2).rxns);
```

```
comparison.unique_rxns_2 = setdiff(models(2).rxns,models(1).rxns);

comparison.common_genes = intersect(models.genes);
comparison.unique_genes_1 = setdiff(models(1).genes,models(2).genes);
comparison.unique_genes_2 = setdiff(models(2).genes,models(1).genes);

comparison.common_TP = intersect(results.TP);
comparison.unique_TP_1 = setdiff(results(1).TP,results(2).TP);
comparison.unique_TP_2 = setdiff(results(2).TP,results(1).TP);

comparison.common_TN = intersect(results.TN);
comparison.unique_TN_1 = setdiff(results(1).TN,results(2).TN);
comparison.unique_TN_2 = setdiff(results(2).TN,results(1).TN);

comparison.common_FP = intersect(results.FP);
comparison.unique_FP_1 = setdiff(results(1).FP,results(2).FP);
comparison.unique_FP_2 = setdiff(results(2).FP,results(1).FP);

comparison.common_FN = intersect(results.FN);
comparison.unique_FN_1 = setdiff(results(1).FN,results(2).FN);
comparison.unique_FN_2 = setdiff(results(2).FN,results(1).FN);

comparison.common_auxotrophs = intersect(results.auxotrophs);
comparison.unique_auxotrophs_1 = setdiff(results(1).auxotrophs, ...
    results(2).auxotrophs);
comparison.unique_auxotrophs_2 = setdiff(results(2).auxotrophs, ...
    results(1).auxotrophs);

%have to calculate which auxotroph predictions are correct
correct_auxotrophs_1 = setdiff(results(1).auxotrophs,...
    union(results(1).auxotroph_viable,results(1).auxotroph_inviable));
correct_auxotrophs_2 = setdiff(results(2).auxotrophs,...
    union(results(2).auxotroph_viable,results(2).auxotroph_inviable));

comparison.common_correct_auxotrophs = intersect(correct_auxotrophs_1, ...
    correct_auxotrophs_2);
comparison.unique_correct_auxotrophs_1 = setdiff(correct_auxotrophs_1, ...
    correct_auxotrophs_2);
comparison.unique_correct_auxotrophs_2 = setdiff(correct_auxotrophs_2, ...
    correct_auxotrophs_1);

comparison.common_auxotrophs_viable = intersect(results.auxotroph_viable);
comparison.unique_auxotrophs_viable_1 = ...
    setdiff(results(1).auxotroph_viable, results(2).auxotroph_viable);
comparison.unique_auxotrophs_viable_2 = ...
    setdiff(results(2).auxotroph_viable, results(1).auxotroph_viable);

comparison.common_auxotrophs_inviable = ...
    intersect(results.auxotroph_inviable);
comparison.unique_auxotrophs_inviable_1 = ...
    setdiff(results(1).auxotroph_inviable,results(2).auxotroph_inviable);
comparison.unique_auxotrophs_inviable_2 = ...
    setdiff(results(2).auxotroph_inviable,results(1).auxotroph_inviable);

%compare constraints
rxnIndexes1 = findRxnIDs(models(1),comparison.common_rxns);
rxnIndexes2 = findRxnIDs(models(2),comparison.common_rxns);

same_lb_indexes = rxnIndexes1(models(1).lb(rxnIndexes1) ==  ...
    models(2).lb(rxnIndexes2));
```

```
differing_lb_indexes = rxnIndexes1(~(models(1).lb(rxnIndexes1) ==  ...
    models(2).lb(rxnIndexes2)));
comparison.same_lb_constraints = models(1).rxns(same_lb_indexes);
comparison.differing_lb_constraints = models(1).rxns(differing_lb_indexes);

same_ub_indexes = rxnIndexes1(models(1).ub(rxnIndexes1) ==  ...
    models(2).ub(rxnIndexes2));
differing_ub_indexes = rxnIndexes1(~(models(1).ub(rxnIndexes1) ==  ...
    models(2).ub(rxnIndexes2)));
comparison.same_ub_constraints = models(1).rxns(same_ub_indexes);
comparison.differing_ub_constraints = models(1).rxns(differing_ub_indexes);
```
