# Supplementary material for: Version 6 of the consensus yeast metabolic network refines biochemical coverage and improves model performance
Source: Database (Oxford). 2013 Aug 9;2013:bat059. doi: 10.1093/database/bat059 (PMC3739857; doi:10.1093/database/bat059)
Supplement: Supplementary Data [file supp_bat059_testYeasteg.pdf]

```
function [results]=testYeast(model, varargin)

% testYeast: a tool for evaluating the Consensus Reconstruction
%
% Input:
%   model     A COBRA Toolbox-format yeast model. Tested with:
%              Yeast 5 - doi:10.1186/1752-0509-6-55
%              Yeast 6 - http://yeast.sf.net (to be published)
%
%   medium (optional)
%             0 = use model-defined medium (default)
%             1 = use minimal medium
%
%   blocked (optional)
%             0 = do not find blocked reactions (default)
%             1 = find blocked reactions (uses FVA, takes longer)
%
%   output (optional)
%             0 = silent
%             1 = default screen output
%             2 = verbose screen output
%
% Output:
%   results   a structure containing the following fields:
%              description = model.description
%              TP = true positive KO predictions (model predicts growth
%                     when nonessential genes are deleted)
%              TN = true negative KO predictions
%              FP = false positive KO predictions
%              FN = false negative KO predictions
%              auxotrophs = auxotroph-inducing genes in model
%              auxotroph_viable = auxotrophs incorrectly predicted to be
%                     viable in miminal media
%              auxotroph_inviable = auxotrophs incorrectly predicted to be
%                     inviable in supplemented media
%              dubious = dubious ORFs in model
%              blocked = blocked reactions in model
%              medium = medium used
%

% modified BH 20 Feb 2013

%% citation
%
% based on testYeastmodel code by kieran smallbone and ben heavner, doi:
% 10.1186/1752-0509-6-55
%
% please cite: Heavner, Benjamin D., Kieran Smallbone, Nathan Price, and
% Larry P. Walker. “Version 6 of the Consensus Yeast Metabolic Network.”
% NEED TO ADD CITATION DETAILS

%% process input arguments
% 3 optional inputs (4 total) at most
numvarargs = length(varargin);
if numvarargs > 3
    error('myfuns:testYeast:TooManyInputs', ...
        'requires at most 3 optional inputs');
end

% set defaults for optional inputs
```

```
optargs = {0 0 1};

% put defaults values into into the valuesToUse cell array,
% and overwrite the ones specified in varargin.
optargs(1:numvarargs) = varargin;

% Place optional args in memorable variable names
[medium blocked output] = optargs{:};

%% set tolerance for KO growth
ko_tol = 1e-6;

%% simple model statistics

if output
    fprintf('\n%s\n\t%s\t%s\n\n\t%g\t%s\n\t%g\t%s\n\t%4g\t%s\n\n',...
        'Model Description:', 'name:', model.description,...
        length(model.mets),'metabolites',...
        length(model.rxns),'reactions',...
        length(model.genes),'genes');
end

%% dubious ORFs

dubiousORFs = setdiff(model.genes,verifiedORFs);

if output
    fprintf('\t%4g\t(%.2f%%)\t%s\n',length(dubiousORFs),...
        (100*length(dubiousORFs)/length(model.genes)),'dubious ORFs');
end

if output == 2
    fprintf('\n\t%s\n','list ORFs included in the model but annotated as dubious by↙
SGD:');
    for k = 1:length(dubiousORFs)
        fprintf('\t\t%s\n',dubiousORFs{k});
    end
end

%% adjust model medium
% ammonium, glucose, oxygen, phosphate, sulphate
%
% note: this isn't a realistic medium (no H+ or H2O for example), but iFF,
% iND, iMM, iAZ, and Y5/6 all predict biomass formation with just these
% components, so it's useful for testing models.

if medium
    if output
        fprintf('\nMaking minimal medium...\n');
    end

    % start with a clean slate: unconstrained excretion, no uptake)
    exchangeRxns = findExcRxns(model);
    model.lb(exchangeRxns) = 0;
    model.ub(exchangeRxns) = 1000;
    desiredExchanges = {...
        'r_1654'; ... % 'ammonium exchange';
        'r_1992'; ... % 'oxygen exchange';
        'r_2005'; ... % 'phosphate exchange';
        'r_2060'; ... % 'sulphate exchange';
```

```
        };
    glucoseExchange = {...
        'r_1714'; ... % D-glucose exchange'
        };
    uptakeRxnIndexes = findRxnIDs(model,desiredExchanges);
    glucoseExchangeIndex = findRxnIDs(model,glucoseExchange);
    if length(uptakeRxnIndexes) ~= 4;
        error('Not all exchange reactions were found.')
    end
    model.lb(uptakeRxnIndexes)=-1000;
    model.lb(glucoseExchangeIndex)=-10;
else
    if output
        fprintf('\nUsing model-default medium...\n');
    end
end

%% create model with complete medium (all exchange reactions switched on)

exchangeRxns = findExcRxns(model);

model_maximal = model;
model_maximal.lb(exchangeRxns) = -1000;
model_maximal.ub(exchangeRxns) = 1000;

%% describe medium
if output
    fprintf('\nCurrent growth medium composition:\n');
    media_indexes = all([exchangeRxns,model.lb ~= 0],2);
    disp([model.rxns(media_indexes) model.rxnNames(media_indexes) ...
        num2cell(model.lb(media_indexes)) num2cell(model.ub(media_indexes))]);
end

%% find blocked reactions (cannot carry any flux)

if blocked
    blockedReactions = findBlockedReaction(model_maximal);

    if output
        fprintf('\n%.0f\t(%.2f%%)\t%s\n',length(blockedReactions),...
            (100*length(blockedReactions)/length(model_maximal.rxns)),...
            'blocked reactions');
    end

    if output == 2
        fprintf('\n\t%s\n','list of blocked reactions:');
        for k = 1:length(blockedReactions)
            fprintf('\t\t%s\n',blockedReactions{k});
        end
    end
end

%% knockout analysis

inviableORFsAll = union(inviableORFs,auxotrophicORFs);
% combine inviable with auxotrophy list - none of these mutants should grow
% in minimal media

exp_retarded = intersect(model.genes,inviableORFsAll);
exp_retarded = intersect(exp_retarded,verifiedORFs);
```

```
exp_viable = setdiff(model.genes,inviableORFsAll);
exp_viable = intersect(exp_viable,verifiedORFs);

grRatio = singleGeneDeletion(model);

mod_viable  = model.genes(grRatio >= ko_tol);
mod_viable = intersect(mod_viable,verifiedORFs);
mod_retarded = model.genes(grRatio < ko_tol);
mod_retarded = intersect(mod_retarded,verifiedORFs);

tp = intersect(exp_viable,mod_viable); n_tp = length(tp);
tn = intersect(exp_retarded,mod_retarded); n_tn = length(tn);
fp = intersect(exp_retarded,mod_viable); n_fp = length(fp);
fn = intersect(exp_viable,mod_retarded); n_fn = length(fn);

if output
    fprintf('\nknockout analysis (positive = viable):');
    fprintf('\n\ttp: %i\n',n_tp);
    fprintf('\ttn: %i\n',n_tn);
    fprintf('\tfp: %i\n',n_fp);
    fprintf('\tfn: %i\n\n',n_fn);
end

n_genes = length(intersect(model.genes,verifiedORFs)); %#ok<NASGU>

sensitivity = (100*n_tp/(n_tp+n_fn));
specificity = (100*n_tn/(n_tn+n_fp));
positivePredictive = (100*n_tp/(n_tp+n_fp));
negativePredictive = (100*n_tn/(n_fn+n_tn));
mcc = (n_tp * n_tn - n_fp * n_fn)/...
    sqrt((n_tp + n_fp)*(n_tp + n_fn)*(n_tn + n_fp)*(n_tn + n_fn));
% geoMean = (sensitivity * specificity)^.5;

if output
    fprintf('\t%.2f%%\t%s\n\t%.2f%%\t%s\n\t%.2f%%\t%s\n\t%.2f%%\t%s\n\n\t%.2f\t%s\n\t\t\t%↙
s\n\n',...
        sensitivity,'sensitivity = recall = tp/(tp+fn)',...
        specificity,'specificity = tn/(tn+fp)',...
        positivePredictive,'positive predictive value = precision = tp/(tp+fp)',...
        negativePredictive,'negative predictive value = tn/(fn+tn)',...
        mcc, 'Matthews correlation coefficient',...
        '(tp*tn-fp*fn)/sqrt((tp+fp)*(tp+fn)*(tn_fp)*(tn+fn))');
end

if output == 2
    fprintf('\tlist of ko false positives:\n');
    for k = 1:n_fp
        fprintf('\t\t%s\n',fp{k});
    end

    fprintf('\n\tlist of ko false negatives:\n');
    for k = 1:n_fn
        fprintf('\t\t%s\n',fn{k});
    end
    disp(' ');
end

%% testing auxotrophy
```

```
% or at least an approximation thereof

auxotrophs = intersect(model.genes,auxotrophicORFs);
auxotrophs = intersect(auxotrophs,verifiedORFs);
n_aux = length(auxotrophs);

grRatio = singleGeneDeletion(model_maximal);

aux_fail = intersect(auxotrophs,mod_viable);

aux_viable  = model.genes(grRatio >= ko_tol);
aux_viable = intersect(aux_viable,auxotrophs);
aux_viable = setdiff(aux_viable,aux_fail);
aux_retarded = model.genes(grRatio < ko_tol);
aux_retarded = intersect(aux_retarded,auxotrophs);
aux_retarded = setdiff(aux_retarded,aux_fail);

if output
    fprintf('auxotrophy analysis:\n');
    fprintf('\t%g\t%s\n\t%g\t[%.2f %%]\t%s\n\t%g\t[%.2f %%]\t%s\n\t%g\t[%.2f %%]\t%
s\n\n',...
        n_aux,'genes',...
        length(aux_viable),(100*length(aux_viable)/n_aux),...
        'correctly predicted',...
        length(aux_fail),(100*length(aux_fail)/n_aux),...
        'viable in minimal medium',...
        length(aux_retarded),(100*length(aux_retarded)/n_aux),...
        'inviable in maximal medium');
end

if output == 2
    fprintf('\tlist of auxotrophs viable in minimal medium:\n');
    for k = 1:length(aux_fail)
        fprintf('\t\t%s\n',aux_fail{k});
    end

    fprintf('\n\tlist of auxotrophs inviable in maximal medium:\n');
    for k = 1:length(aux_retarded)
        fprintf('\t\t%s\n',aux_retarded{k});
    end
end

if output
    fprintf('==\n\n');
end

%% report results
results.description = model.description;
results.TP = tp;
results.TN = tn;
results.FP = fp;
results.FN = fn;
results.auxotrophs = auxotrophs;
results.auxotroph_viable = aux_fail;
results.auxotroph_inviable = aux_retarded;
results.dubious = dubiousORFs;
if exist('blockedReactions')
    results.blocked = blockedReactions;
end
if medium
```

```
    results.medium = 'minimal medium';
else
    results.medium = 'model default medium';
end

end

%% required functions

function blockedReactions = findBlockedReaction(model)
%findBlockedReaction determines those reactions which cannot carry any
%flux in the given simulation conditions.
%
% BlockedReaction = findBlockedReaction(model)
%
%INPUT
% model               COBRA model structure
%
%OUTPUT
% blockedReactions    List of blocked reactions
%
%
% Ines Thiele 02/09
% kieran: 15 aug 11
% James Eddy: 19 Feb 13

tol = 1e-10;
% << changed by ks
% blockedReactions =[];
%blockedReactions = {};
% >>
[minMax(:,1),minMax(:,2)] = fluxVariability(model,0);
%cnt = 1;
%for i=1:length(minMax)
%    if (minMax(i,2) < tol && minMax(i,2) > -tol && minMax(i,1) < tol && minMax(i,1) > -↙
tol)
%        blockedReactions(cnt) = model.rxns(i); %#ok<AGROW>
%        cnt = cnt + 1;
%    end
%end

blocked = abs(minMax(:,1)) < tol & abs(minMax(:,2)) < tol;
blockedReactions = model.rxns(blocked);

end

%% gene lists
% BH is Ben Heavner (bheavner@gmail.com), HA is Hnin Aung
% (hnin.w.aung@gmail.com)
function genes = inviableORFs

% list of 1192 unique inviable ORFs taken from the Yeast Deletion Project (14 aug
% 11)
% http://www-sequence.stanford.edu/group/yeast_deletion_project/downloads.html

genes = {'YAL001C';'YAL003W';'YAL025C';'YAL032C';'YAL033W';'YAL034W-a';'YAL035C-↙
A';'YAL038W';'YAL041W';'YAL043C';'YAR007C';'YAR008W';'YAR019C';'YBL004W';'YBL014C';'YBL018↙
C';'YBL020W';'YBL023C';'YBL026W';'YBL030C';'YBL034C';'YBL035C';'YBL040C';'YBL041W';'YBL050↙
W';'YBL073W';'YBL074C';'YBL076C';'YBL077W';'YBL084C';'YBL092W';'YBL097W';'YBL105C';'YBR002↙
C';'YBR004C';'YBR011C';'YBR029C';'YBR038W';'YBR049C';'YBR055C';'YBR060C';'YBR070C';'YBR079↙
```

```
C';'YBR080C';'YBR087W';'YBR087W';'YBR088C';'YBR089W';'YBR091C';'YBR102C';'YBR109C';'YBR110↙
W';'YBR123C';'YBR124W';'YBR135W';'YBR136W';'YBR140C';'YBR142W';'YBR143C';'YBR152W';'YBR153↙
W';'YBR154C';'YBR155W';'YBR160W';'YBR167C';'YBR190W';'YBR192W';'YBR193C';'YBR196C';'YBR198↙
C';'YBR202W';'YBR211C';'YBR233W-A';'YBR233W-↙
A';'YBR234C';'YBR236C';'YBR237W';'YBR243C';'YBR247C';'YBR252W';'YBR253W';'YBR254C';'YBR256↙
C';'YBR257W';'YBR265W';'YCL003W';'YCL017C';'YCL031C';'YCL031C';'YCL031C';'YCL043C';'YCL052↙
C';'YCL053C';'YCL054W';'YCL059C';'YCR012W';'YCR012W';'YCR013C';'YCR013C';'YCR035C';'YCR052↙
W';'YCR054C';'YCR057C';'YCR072C';'YCR093W';'YDL003W';'YDL004W';'YDL007W';'YDL008W';'YDL014↙
W';'YDL015C';'YDL016C';'YDL017W';'YDL028C';'YDL029W';'YDL030W';'YDL031W';'YDL043C';'YDL045↙
C';'YDL055C';'YDL058W';'YDL060W';'YDL064W';'YDL084W';'YDL087C';'YDL092W';'YDL097C';'YDL098↙
C';'YDL102W';'YDL103C';'YDL105W';'YDL108W';'YDL111C';'YDL120W';'YDL126C';'YDL132W';'YDL139↙
C';'YDL140C';'YDL141W';'YDL143W';'YDL145C';'YDL147W';'YDL148C';'YDL150W';'YDL152W';'YDL153↙
C';'YDL163W';'YDL164C';'YDL165W';'YDL166C';'YDL193W';'YDL195W';'YDL196W';'YDL205C';'YDL207↙
W';'YDL208W';'YDL209C';'YDL212W';'YDL217C';'YDL220C';'YDL221W';'YDL235C';'YDR002W';'YDR013↙
W';'YDR016C';'YDR021W';'YDR023W';'YDR037W';'YDR041W';'YDR044W';'YDR045C';'YDR047W';'YDR050↙
C';'YDR050C';'YDR052C';'YDR053W';'YDR054C';'YDR060W';'YDR062W';'YDR064W';'YDR081C';'YDR082↙
W';'YDR086C';'YDR087C';'YDR088C';'YDR091C';'YDR113C';'YDR118W';'YDR141C';'YDR145W';'YDR160↙
W';'YDR164C';'YDR166C';'YDR167W';'YDR168W';'YDR170C';'YDR172W';'YDR177W';'YDR180W';'YDR182↙
W';'YDR187C';'YDR188W';'YDR189W';'YDR190C';'YDR196C';'YDR201W';'YDR208W';'YDR211W';'YDR212↙
W';'YDR224C';'YDR224C';'YDR224C';'YDR228C';'YDR232W';'YDR235W';'YDR236C';'YDR238C';'YDR240↙
C';'YDR243C';'YDR246W';'YDR267C';'YDR280W';'YDR288W';'YDR292C';'YDR299W';'YDR301W';'YDR302↙
W';'YDR303C';'YDR308C';'YDR311W';'YDR320C-A';'YDR320C-↙
A';'YDR324C';'YDR325W';'YDR327W';'YDR328C';'YDR331W';'YDR339C';'YDR341C';'YDR353W';'YDR355↙
C';'YDR356W';'YDR361C';'YDR362C';'YDR365C';'YDR367W';'YDR373W';'YDR376W';'YDR381W';'YDR390↙
C';'YDR394W';'YDR396W';'YDR397C';'YDR398W';'YDR404C';'YDR407C';'YDR412W';'YDR413C';'YDR416↙
W';'YDR427W';'YDR427W';'YDR429C';'YDR434W';'YDR437W';'YDR449C';'YDR454C';'YDR460W';'YDR464↙
W';'YDR468C';'YDR472W';'YDR473C';'YDR478W';'YDR487C';'YDR489W';'YDR498C';'YDR499W';'YDR510↙
W';'YDR526C';'YDR527W';'YDR531W';'YEL002C';'YEL019C';'YEL026W';'YEL032W';'YEL034W';'YEL035↙
C';'YEL055C';'YEL058W';'YER003C';'YER006W';'YER008C';'YER009W';'YER012W';'YER013W';'YER018↙
C';'YER021W';'YER022W';'YER023W';'YER025W';'YER029C';'YER029C';'YER036C';'YER038C';'YER043↙
C';'YER048W-A';'YER074W-A';'YER074W-↙
A';'YER082C';'YER093C';'YER094C';'YER104W';'YER112W';'YER125W';'YER126C';'YER127W';'YER133↙
W';'YER136W';'YER146W';'YER147C';'YER148W';'YER157W';'YER159C';'YER165W';'YER168C';'YER171↙
W';'YER172C';'YFL002C';'YFL005W';'YFL008W';'YFL009W';'YFL017C';'YFL018W-↙
A';'YFL022C';'YFL024C';'YFL029C';'YFL035C';'YFL035C-↙
A';'YFL037W';'YFL038C';'YFL039C';'YFL045C';'YFR002W';'YFR003C';'YFR004W';'YFR005C';'YFR027↙
W';'YFR028C';'YFR029W';'YFR031C';'YFR037C';'YFR042W';'YFR050C';'YFR051C';'YFR052W';'YGL001↙
C';'YGL008C';'YGL011C';'YGL018C';'YGL022W';'YGL030W';'YGL040C';'YGL044C';'YGL044C';'YGL044↙
C';'YGL047W';'YGL048C';'YGL055W';'YGL061C';'YGL065C';'YGL068W';'YGL069C';'YGL073W';'YGL074↙
C';'YGL075C';'YGL091C';'YGL092W';'YGL093W';'YGL097W';'YGL097W';'YGL098W';'YGL099W';'YGL102↙
C';'YGL103W';'YGL111W';'YGL112C';'YGL113W';'YGL116W';'YGL120C';'YGL122C';'YGL123W';'YGL128↙
C';'YGL130W';'YGL137W';'YGL142C';'YGL145W';'YGL145W';'YGL145W';'YGL150C';'YGL155W';'YGL169↙
W';'YGL171W';'YGL172W';'YGL201C';'YGL207W';'YGL225W';'YGL233W';'YGL238W';'YGL239C';'YGL239↙
C';'YGL245W';'YGL247W';'YGR002C';'YGR005C';'YGR009C';'YGR013W';'YGR024C';'YGR029W';'YGR029↙
W';'YGR029W';'YGR030C';'YGR046W';'YGR047C';'YGR048W';'YGR060W';'YGR065C';'YGR073C';'YGR074↙
W';'YGR075C';'YGR082W';'YGR083C';'YGR090W';'YGR091W';'YGR094W';'YGR095C';'YGR098C';'YGR099↙
W';'YGR103W';'YGR113W';'YGR114C';'YGR115C';'YGR116W';'YGR119C';'YGR120C';'YGR128C';'YGR140↙
W';'YGR145W';'YGR147C';'YGR156W';'YGR158C';'YGR172C';'YGR175C';'YGR179C';'YGR185C';'YGR186↙
W';'YGR190C';'YGR191W';'YGR195W';'YGR198W';'YGR211W';'YGR216C';'YGR218W';'YGR245C';'YGR246↙
C';'YGR251W';'YGR253C';'YGR264C';'YGR265W';'YGR267C';'YGR274C';'YGR277C';'YGR278W';'YGR280↙
C';'YHL015W';'YHR005C-↙
A';'YHR007C';'YHR019C';'YHR020W';'YHR023W';'YHR024C';'YHR036W';'YHR040W';'YHR042W';'YHR058↙
C';'YHR062C';'YHR065C';'YHR068W';'YHR069C';'YHR070W';'YHR072W';'YHR072W-A';'YHR072W-↙
A';'YHR074W';'YHR083W';'YHR085W';'YHR088W';'YHR088W';'YHR088W';'YHR089C';'YHR089C';'YHR101↙
C';'YHR102W';'YHR102W';'YHR107C';'YHR118C';'YHR122W';'YHR128W';'YHR128W';'YHR143W-↙
A';'YHR148W';'YHR164C';'YHR165C';'YHR165C';'YHR166C';'YHR169W';'YHR169W';'YHR170W';'YHR172↙
W';'YHR186C';'YHR188C';'YHR188C';'YHR188C';'YHR190W';'YHR196W';'YHR197W';'YHR197W';'YHR197↙
W';'YHR199C-A';'YHR199C-↙
A';'YIL003W';'YIL004C';'YIL019W';'YIL021W';'YIL022W';'YIL026C';'YIL031W';'YIL046W';'YIL048↙
W';'YIL051C';'YIL061C';'YIL062C';'YIL063C';'YIL068C';'YIL075C';'YIL078W';'YIL083C';'YIL091↙
```

```
C';'YIL104C';'YIL106W';'YIL106W';'YIL106W';'YIL109C';'YIL115C';'YIL118W';'YIL126W';'YIL129
C';'YIL142W';'YIL143C';'YIL144W';'YIL147C';'YIL150C';'YIL171W';'YIR006C';'YIR008C';'YIR010
W';'YIR011C';'YIR012W';'YIR015W';'YIR022W';'YJL001W';'YJL002C';'YJL005W';'YJL008C';'YJL008
C';'YJL008C';'YJL009W';'YJL010C';'YJL011C';'YJL014W';'YJL015C';'YJL018W';'YJL019W';'YJL025
W';'YJL026W';'YJL031C';'YJL032W';'YJL033W';'YJL034W';'YJL035C';'YJL039C';'YJL041W';'YJL050
W';'YJL054W';'YJL061W';'YJL069C';'YJL072C';'YJL074C';'YJL076W';'YJL081C';'YJL085W';'YJL086
C';'YJL087C';'YJL090C';'YJL091C';'YJL097W';'YJL104W';'YJL109C';'YJL111W';'YJL125C';'YJL143
W';'YJL156C';'YJL167W';'YJL173C';'YJL174W';'YJL194W';'YJL195C';'YJL195C';'YJL202C';'YJL202
C';'YJL203W';'YJR002W';'YJR006W';'YJR007W';'YJR012C';'YJR013W';'YJR016C';'YJR017C';'YJR022
W';'YJR023C';'YJR041C';'YJR042W';'YJR045C';'YJR046W';'YJR046W';'YJR046W';'YJR057W';'YJR064
W';'YJR065C';'YJR067C';'YJR068W';'YJR072C';'YJR076C';'YJR089W';'YJR089W';'YJR093C';'YJR112
W';'YJR123W';'YJR141W';'YKL004W';'YKL006C-
A';'YKL012W';'YKL013C';'YKL014C';'YKL018W';'YKL019W';'YKL021C';'YKL022C';'YKL024C';'YKL028
W';'YKL033W';'YKL035W';'YKL036C';'YKL042W';'YKL045W';'YKL049C';'YKL049C';'YKL049C';'YKL052
C';'YKL058W';'YKL059C';'YKL060C';'YKL078W';'YKL082C';'YKL083W';'YKL088W';'YKL089W';'YKL095
W';'YKL099C';'YKL104C';'YKL108W';'YKL111C';'YKL112W';'YKL122C';'YKL125W';'YKL138C-
A';'YKL138C-
A';'YKL141W';'YKL144C';'YKL145W';'YKL152C';'YKL153W';'YKL154W';'YKL165C';'YKL172W';'YKL172
W';'YKL172W';'YKL173W';'YKL180W';'YKL182W';'YKL186C';'YKL189W';'YKL193C';'YKL195W';'YKL196
C';'YKL203C';'YKL210W';'YKR002W';'YKR004C';'YKR008W';'YKR022C';'YKR025W';'YKR037C';'YKR038
C';'YKR062W';'YKR063C';'YKR068C';'YKR071C';'YKR079C';'YKR081C';'YKR083C';'YKR086W';'YLL003
W';'YLL004W';'YLL008W';'YLL011W';'YLL018C';'YLL031C';'YLL034C';'YLL035W';'YLL036C';'YLL037
W';'YLL050C';'YLR002C';'YLR005W';'YLR007W';'YLR008C';'YLR009W';'YLR010C';'YLR022C';'YLR026
C';'YLR029C';'YLR033W';'YLR045C';'YLR051C';'YLR060W';'YLR066W';'YLR071C';'YLR075W';'YLR076
C';'YLR078C';'YLR086W';'YLR088W';'YLR099W-A';'YLR099W-
A';'YLR100W';'YLR101C';'YLR103C';'YLR105C';'YLR106C';'YLR115W';'YLR116W';'YLR117C';'YLR127
C';'YLR129W';'YLR132C';'YLR140W';'YLR141W';'YLR145W';'YLR147C';'YLR153C';'YLR163C';'YLR166
C';'YLR167W';'YLR175W';'YLR186W';'YLR186W';'YLR195C';'YLR196W';'YLR197W';'YLR198C';'YLR208
W';'YLR212C';'YLR215C';'YLR222C';'YLR223C';'YLR229C';'YLR230W';'YLR243W';'YLR249W';'YLR259
C';'YLR272C';'YLR274W';'YLR275W';'YLR276C';'YLR277C';'YLR291C';'YLR293C';'YLR298C';'YLR305
C';'YLR310C';'YLR314C';'YLR316C';'YLR316C';'YLR316C';'YLR317W';'YLR321C';'YLR323C';'YLR336
C';'YLR339C';'YLR340W';'YLR347C';'YLR355C';'YLR359W';'YLR378C';'YLR379W';'YLR383W';'YLR397
C';'YLR409C';'YLR424W';'YLR430W';'YLR438C-
A';'YLR440C';'YLR457C';'YLR458W';'YLR459W';'YML010W';'YML015C';'YML015C';'YML023C';'YML023
C';'YML025C';'YML031W';'YML043C';'YML046W';'YML049C';'YML064C';'YML065W';'YML069W';'YML077
W';'YML085C';'YML091C';'YML092C';'YML092C';'YML092C';'YML093W';'YML098W';'YML105C';'YML114
C';'YML125C';'YML126C';'YML127W';'YML130C';'YMR001C';'YMR005W';'YMR005W';'YMR013C';'YMR028
W';'YMR033W';'YMR033W';'YMR043W';'YMR047C';'YMR047C';'YMR049C';'YMR059W';'YMR059W';'YMR059
W';'YMR061W';'YMR076C';'YMR079W';'YMR093W';'YMR094W';'YMR108W';'YMR108W';'YMR112C';'YMR113
W';'YMR117C';'YMR128W';'YMR131C';'YMR134W';'YMR146C';'YMR149W';'YMR168C';'YMR197C';'YMR200
W';'YMR203W';'YMR208W';'YMR211W';'YMR213W';'YMR218C';'YMR220W';'YMR227C';'YMR229C';'YMR235
C';'YMR236W';'YMR239C';'YMR240C';'YMR260C';'YMR268C';'YMR270C';'YMR277W';'YMR281W';'YMR288
W';'YMR290C';'YMR290W-
A';'YMR296C';'YMR298W';'YMR301C';'YMR308C';'YMR309C';'YMR314W';'YNL002C';'YNL006W';'YNL007
C';'YNL024C-A';'YNL024C-
A';'YNL026W';'YNL036W';'YNL036W';'YNL038W';'YNL039W';'YNL061W';'YNL062C';'YNL075W';'YNL088
W';'YNL102W';'YNL103W';'YNL110C';'YNL112W';'YNL112W';'YNL112W';'YNL113W';'YNL114C';'YNL118
C';'YNL124W';'YNL126W';'YNL126W';'YNL131W';'YNL132W';'YNL137C';'YNL138W-A';'YNL138W-
A';'YNL149C';'YNL150W';'YNL151C';'YNL152W';'YNL158W';'YNL161W';'YNL163C';'YNL172W';'YNL178
W';'YNL181W';'YNL182C';'YNL188W';'YNL189W';'YNL207W';'YNL216W';'YNL221C';'YNL222W';'YNL232
W';'YNL240C';'YNL244C';'YNL245C';'YNL247W';'YNL251C';'YNL256W';'YNL258C';'YNL260C';'YNL261
W';'YNL262W';'YNL263C';'YNL267W';'YNL272C';'YNL282W';'YNL287W';'YNL290W';'YNL306W';'YNL308
C';'YNL310C';'YNL312W';'YNL313C';'YNL317W';'YNR003C';'YNR011C';'YNR016C';'YNR017W';'YNR026
C';'YNR035C';'YNR038W';'YNR043W';'YNR046W';'YNR053C';'YNR054C';'YOL005C';'YOL010W';'YOL021
C';'YOL022C';'YOL026C';'YOL034W';'YOL038W';'YOL040C';'YOL066C';'YOL069W';'YOL077C';'YOL078
W';'YOL094C';'YOL097C';'YOL102C';'YOL120C';'YOL123W';'YOL127W';'YOL130W';'YOL133W';'YOL134
C';'YOL135C';'YOL139C';'YOL142W';'YOL142W';'YOL144W';'YOL146W';'YOL149W';'YOR004W';'YOR020
C';'YOR046C';'YOR048C';'YOR056C';'YOR057W';'YOR060C';'YOR063W';'YOR074C';'YOR075W';'YOR077
W';'YOR095C';'YOR098C';'YOR102W';'YOR103C';'YOR110W';'YOR116C';'YOR117W';'YOR119C';'YOR122
C';'YOR143C';'YOR145C';'YOR146W';'YOR148C';'YOR149C';'YOR151C';'YOR157C';'YOR159C';'YOR160
```

```
W';'YOR168W';'YOR169C';'YOR174W';'YOR176W';'YOR181W';'YOR194C';'YOR203W';'YOR204W';'YOR206↙
W';'YOR207C';'YOR210W';'YOR217W';'YOR218C';'YOR224C';'YOR232W';'YOR236W';'YOR244W';'YOR249↙
C';'YOR250C';'YOR254C';'YOR256C';'YOR257W';'YOR259C';'YOR260W';'YOR261C';'YOR262W';'YOR272↙
W';'YOR278W';'YOR281C';'YOR282W';'YOR287C';'YOR294W';'YOR310C';'YOR319W';'YOR326W';'YOR329↙
C';'YOR335C';'YOR336W';'YOR340C';'YOR341W';'YOR353C';'YOR361C';'YOR362C';'YOR370C';'YOR372↙
C';'YOR373W';'YPL007C';'YPL010W';'YPL011C';'YPL012W';'YPL016W';'YPL020C';'YPL028W';'YPL043↙
W';'YPL044C';'YPL063W';'YPL076W';'YPL082C';'YPL083C';'YPL085W';'YPL093W';'YPL094C';'YPL117↙
C';'YPL122C';'YPL124W';'YPL126W';'YPL128C';'YPL131W';'YPL142C';'YPL143W';'YPL146C';'YPL151↙
C';'YPL153C';'YPL160W';'YPL169C';'YPL175W';'YPL190C';'YPL204W';'YPL209C';'YPL210C';'YPL211↙
W';'YPL217C';'YPL218W';'YPL228W';'YPL231W';'YPL233W';'YPL235W';'YPL237W';'YPL238C';'YPL242↙
C';'YPL243W';'YPL251W';'YPL252C';'YPL255W';'YPL266W';'YPR010C';'YPR016C';'YPR019W';'YPR025↙
C';'YPR033C';'YPR034W';'YPR035W';'YPR041W';'YPR048W';'YPR055W';'YPR056W';'YPR082C';'YPR085↙
C';'YPR086W';'YPR088C';'YPR094W';'YPR103W';'YPR104C';'YPR105C';'YPR107C';'YPR108W';'YPR110↙
C';'YPR112C';'YPR113W';'YPR133C';'YPR136C';'YPR137W';'YPR142C';'YPR143W';'YPR144C';'YPR161↙
C';'YPR162C';'YPR165W';'YPR168W';'YPR169W';'YPR175W';'YPR176C';'YPR177C';'YPR178W';'YPR180↙
W';'YPR181C';'YPR182W';'YPR183W';'YPR186C';'YPR187W';'YPR190C';...
    %'YCL004W';... %removed by HA 2/7/2013 - doi: 10.1074/jbc.273.16.9829
    %'YKL192C';... %removed by HA 2/7/2013 - http://dx.doi.org/10.1016/j.bbalip.↙
2006.07.004
    };

end

function genes = auxotrophicORFs

% list of 432 auxotrophic ORFs (669 annotated auxotroph minus 52 on YKO
% essential list minus 87 which are re either temp-sensitive ino
% auxotrophs or not auxotrophs in the nature study):

genes=↙
{'YAL021C';'YAL024C';'YAL026C';'YAL040C';'YAL051W';'YAL056W';'YAL058W';'YAR003W';'YAR015W'↙
;'YAR069W-A';'YAR070W-A';'YBL027W';'YBL033C';'YBL047C';'YBL058W';'YBL061C';'YBL091C-↙
A';'YBL102W';'YBL103C';'YBR015C';'YBR058C';'YBR077C';'YBR106W';'YBR107C';'YBR115C';'YBR126↙
C';'YBR127C';'YBR133C';'YBR175W';'YBR176W';'YBR189W';'YBR191W';'YBR248C';'YBR272C';'YBR279↙
W';'YCL018W';'YCL030C';'YCL032W';'YCL033C';'YCL045C';'YCR021C';'YCR045C';'YCR047C';'YCR053↙
W';'YCR076C';'YCR089W';'YCR094W';'YDL001W';'YDL002C';'YDL006W';'YDL010W';'YDL020C';'YDL021↙
W';'YDL033C';'YDL040C';'YDL048C';'YDL069C';'YDL073W';'YDL074C';'YDL077C';'YDL081C';'YDL083↙
C';'YDL106C';'YDL130W';'YDL173W';'YDL190C';'YDL191W';'YDL192W';'YDL194W';'YDL201W';'YDL203↙
C';'YDR007W';'YDR043C';'YDR049W';'YDR057W';'YDR071C';'YDR074W';'YDR080W';'YDR138W';'YDR162↙
C';'YDR173C';'YDR174W';'YDR176W';'YDR200C';'YDR226W';'YDR260C';'YDR266C';'YDR276C';'YDR277↙
C';'YDR283C';'YDR289C';'YDR335W';'YDR346C';'YDR348C';'YDR351W';'YDR354W';'YDR358W';'YDR363↙
W';'YDR379W';'YDR385W';'YDR389W';'YDR392W';'YDR395W';'YDR411C';'YDR422C';'YDR432W';'YDR439↙
W';'YDR448W';'YDR469W';'YDR477W';'YDR482C';'YDR486C';'YDR540C';'YEL004W';'YEL013W';'YEL021↙
W';'YEL027W';'YEL031W';'YEL037C';'YEL040W';'YEL044W';'YEL048C';'YEL051W';'YER001W';'YER007↙
C-↙
A';'YER026C';'YER027C';'YER052C';'YER055C';'YER059W';'YER069W';'YER083C';'YER090W';'YER091↙
C';'YER092W';'YER095W';'YER101C';'YER116C';'YER118C';'YER120W';'YER122C';'YER129W';'YER130↙
C';'YER149C';'YER150W';'YER167W';'YER169W';'YER177W';'YFL013C';'YFL031W';'YFR010W';'YFR025↙
C';'YFR040W';'YFR048W';'YGL009C';'YGL012W';'YGL020C';'YGL025C';'YGL026C';'YGL031C';'YGL049↙
C';'YGL054C';'YGL058W';'YGL060W';'YGL066W';'YGL070C';'YGL115W';'YGL126W';'YGL127C';'YGL154↙
C';'YGL167C';'YGL168W';'YGL175C';'YGL179C';'YGL180W';'YGL181W';'YGL203C';'YGL211W';'YGL219↙
C';'YGL234W';'YGL244W';'YGR014W';'YGR020C';'YGR056W';'YGR057C';'YGR061C';'YGR063C';'YGR092↙
W';'YGR104C';'YGR105W';'YGR108W';'YGR135W';'YGR144W';'YGR162W';'YGR166W';'YGR204W';'YGR223↙
C';'YGR227W';'YGR229C';'YGR241C';'YGR250C';'YGR252W';'YHR010W';'YHR013C';'YHR018C';'YHR025↙
W';'YHR026W';'YHR030C';'YHR038W';'YHR060W';'YHR079C';'YHR111W';'YHR142W';'YHR162W';'YHR178↙
W';'YHR179W';'YHR199C';'YHR206W';'YHR208W';'YIL017C';'YIL020C';'YIL027C';'YIL029C';'YIL036↙
W';'YIL044C';'YIL072W';'YIL077C';'YIL105C';'YIL116W';'YIL119C';'YIL128W';'YIL153W';'YIR017↙
C';'YIR034C';'YJL088W';'YJL095W';'YJL115W';'YJL117W';'YJL128C';'YJL130C';'YJL140W';'YJL151↙
C';'YJL153C';'YJL158C';'YJL172W';'YJL192C';'YJL193W';'YJL204C';'YJL208C';'YJR066W';'YJR075↙
W';'YJR083C';'YJR104C';'YJR122W';'YKL001C';'YKL006W';'YKL027W';'YKL032C';'YKL048C';'YKL053↙
```

```
C-
A';'YKL056C';'YKL064W';'YKL077W';'YKL079W';'YKL119C';'YKL121W';'YKL160W';'YKL176C';'YKL190
W';'YKL211C';'YKL213C';'YKL216W';'YKR007W';'YKR026C';'YKR036C';'YKR070W';'YKR099W';'YLL019
C';'YLL021W';'YLL027W';'YLL039C';'YLR015W';'YLR016C';'YLR021W';'YLR048W';'YLR055C';'YLR061
W';'YLR074C';'YLR079W';'YLR087C';'YLR113W';'YLR150W';'YLR192C';'YLR199C';'YLR226W';'YLR242
C';'YLR262C';'YLR268W';'YLR292C';'YLR315W';'YLR320W';'YLR324W';'YLR332W';'YLR357W';'YLR371
W';'YLR373C';'YLR396C';'YLR417W';'YLR418C';'YLR420W';'YLR426W';'YLR436C';'YML008C';'YML013
W';'YML014W';'YML028W';'YML034W';'YML055W';'YML071C';'YML103C';'YML115C';'YML117W';'YMR010
W';'YMR014W';'YMR016C';'YMR029C';'YMR038C';'YMR052W';'YMR062C';'YMR067C';'YMR068W';'YMR092
C';'YMR099C';'YMR104C';'YMR123W';'YMR190C';'YMR202W';'YMR214W';'YMR217W';'YMR242C';'YMR247
C';'YMR276W';'YMR300C';'YMR304W';'YMR307W';'YMR312W';'YNL003C';'YNL041C';'YNL051W';'YNL079
C';'YNL080C';'YNL119W';'YNL127W';'YNL133C';'YNL148C';'YNL215W';'YNL219C';'YNL220W';'YNL229
C';'YNL236W';'YNL277W';'YNL307C';'YNL316C';'YNL322C';'YNR055C';'YOL018C';'YOL067C';'YOL087
C';'YOL090W';'YOL093W';'YOL098C';'YOL107W';'YOL109W';'YOL111C';'YOL116W';'YOL121C';'YOL122
C';'YOL124C';'YOL143C';'YOL145C';'YOR002W';'YOR008C';'YOR012W';'YOR067C';'YOR070C';'YOR078
W';'YOR096W';'YOR106W';'YOR123C';'YOR128C';'YOR189W';'YOR202W';'YOR216C';'YOR246C';'YOR290
C';'YOR320C';'YOR322C';'YOR359W';'YOR371C';'YPL055C';'YPL065W';'YPL089C';'YPL138C';'YPL140
C';'YPL144W';'YPL157W';'YPL159C';'YPL174C';'YPL177C';'YPL226W';'YPL241C';'YPL254W';'YPL264
C';'YPR036W';'YPR043W';'YPR060C';'YPR067W';'YPR139C';'YPR167C';'YPR173C';'YPR179C';'YPR201
W';...
    'YDR158W';... %added by BH 10/10/12 from SGD annotation
    'YDR408C';... %added by BH 10/10/12 from SGD annotation
    'YFR030W';... %added by BH 10/10/12 from SGD annotation
    'YJR137C';... %added by BH 10/10/12 from SGD annotation
    %'YBL098W';... %removed by BH 10/11/12 - per SGD, auxotroph in W303, not S288C
    %'YDL131W';... %removed by BH 10/11/12 - per SGD, auxotrophy is only observed during
respiratory growth in non S288C strain
    %'YFR047C';... %removed by BH 10/11/12 - per SGD, auxotroph in W303, not S288C
    %'YKL212W';... %removed by BH 10/11/12 - per SGD, auxotroph not in S288C
    %'YNL241C';... %removed by BH 10/11/12 - per SGD, auxotroph not in S288C
    'YDR234W';... %added by BH 10/15/12 - based on PMID: 5908136 - poor evidence, and
2507177. I am gaining confidence that amino acid defects are masked in YKO strains by
auxotrophy (similarly, I suspect "false negatives" YBR166C, YDR127W, YGL148W, YIL094C,
YOL140W (?))
    'YJR139C';... %added by BH 10/15/12 - based on PMID: 4380684 - as above, poor
evidence, but reasonable
    'YNL280C';... %added by BH 10/15/12 - this one is quite a puzzle. Grows aerobically on
defined media, but not complex media. See PMID 8631695
    'YNR050C';... %added by bh 10/15/12 - PMID 6429126 describes it as a lysine auxotroph
(not sure if strain specific)
    'YOL058W';... %added by bh 10/15/12 - PMID 19346356 "strains Arg1, Arg3, and Arg4 are
arginine auxotrophic due to defects in arginine biosynthesis"
%    'YDR300C';... %added by bh 10/15/12 - per SGD, pro1, pro2, pro3 are auxotrophs
however: "A unique property of all the pro mutant strains is that they cannot grow on
standard YPD rich media."
%    'YOR323C';... %added by bh 10/15/12 - per SGD, pro1, pro2, pro3 are auxotrophs
however: "A unique property of all the pro mutant strains is that they cannot grow on
standard YPD rich media."
%    'YER023W';...%added by bh 10/15/12 - per SGD, pro1, pro2, pro3 are auxotrophs
however: "A unique property of all the pro mutant strains is that they cannot grow on
standard YPD rich media."
    'YIL094C';... %added by bh 10/26/12 - PMID 6026248
    'YGL080W';... %added by bh 11/1/12 - PMID 22628554, a leucine and valine auxotroph
    'YHR162W';... %added by bh 11/1/12 - PMID 22628554, a leucine and valine auxotroph
    'YMR289W';... %added by bh 12/7/12 - PMID 17873082, required for growth in media
lacking p-aminobenzoic or folic acid and encoded a 4-amino-4-deoxychorismate lyase, which
is the last of the three enzymatic activities required for p-aminobenzoic acid
biosynthesis
    'YAL012W';... %added by bh 12/7/12 - per SGD, cysteine auxotroph
    'YER086W';... %added by bh 12/7/12 - per SGD, isoleucine auxotroph
```

```
    'YGR155W';... %added by bh 12/7/12 - per SGD, cysteine auxotroph
    'YLR304C';... %added by bh 12/7/12 - per SGD, mutation leads to glutamate auxotrophy
    'YBR252W';... %added by bh 12/11/12 - per SGD, mutation leads to dTMP auxotrophy
    'YDL205C';... %added by bh 12/11/12 - per SGD, mutation leads to heme auxotrophy
    'YDR047W';... %added by bh 12/11/12 - per SGD, mutation leads to heme auxotrophy
    'YDR232W';... %added by bh 12/11/12 - per SGD, mutation leads to heme auxotrophy
    %'YFR019W';... %removed by BH 12/11/12 - temp sensitive inositol auxotroph
    'YGL040C';... %added by bh 12/11/12 - per SGD, mutation leads to heme auxotrophy
    %'YGL062W';... %removed by BH 12/11/12 - temp sensitive inositol auxotroph
    'YGR065C';... %added by bh 12/11/12 - per SGD, mutation leads to fatty acid auxotrophy
    'YGR267C';... %added by bh 12/11/12 - per SGD, mutation leads to folinic acid
auxotrophy
    'YHR128W';... %added by bh 12/11/12 - per SGD, mutation leads to uracil and uridine
auxotrophy
    'YMR113W';... %added by bh 12/11/12 - per SGD, mutation leads to folinic acid
auxotrophy
    'YNL256W';... %added by bh 12/11/12 - per SGD, mutation leads to folic acid auxotrophy
    'YOR278W';... %added by bh 12/11/12 - per SGD, mutation leads to heme auxotrophy
    'YGL055W';... %added by bh 12/13/12 - per SGD, mutation leads to oleic acid auxotrophy
(probably monounsaturated fatty acids in general)
    %'YER152C';... %removed by BH 1/8/13 - temp sensitive inositol auxotroph
    %'YMR165C';... %removed by HA 2/5/13 - auxotrophy is due to overexpression, not
deletion.
    %'YEL029C';... %removed by bh 2/5/13 - temp dependent inositol auxotroph
    'YBR166C';... %added by bh 2/7/13 - tyrosine auxotroph PMID: 1943992 - ref
surprisingly hard to find, since the gene is "TYrosine Requiring"
    'YLR303W';... %added by bh 2/7/13 - methionine auxotroph PMIDs: 4364332, 1101032
    };
end

function genes = verifiedORFs

% list of 4992 verified ORFs taken from SGD (6 Feb 2013)
% http://www.yeastgenome.org/cgi-bin/search/featureSearch?
featuretype=ORF&qualifier=Verified
% Note: this still includes some ORFs with "dubious" function

genes =
{'Q0045';'Q0050';'Q0055';'Q0060';'Q0065';'Q0070';'Q0080';'Q0085';'Q0105';'Q0110';'Q0115';'
Q0120';'Q0130';'Q0140';'Q0160';'Q0250';'Q0275';'R0010W';'R0020C';'R0030W';'R0040C';'YAL001
C';'YAL002W';'YAL003W';'YAL005C';'YAL007C';'YAL008W';'YAL009W';'YAL010C';'YAL011W';'YAL012
W';'YAL013W';'YAL014C';'YAL015C';'YAL016W';'YAL017W';'YAL019W';'YAL020C';'YAL021C';'YAL022
C';'YAL023C';'YAL024C';'YAL025C';'YAL026C';'YAL027W';'YAL028W';'YAL029C';'YAL030W';'YAL031
C';'YAL032C';'YAL033W';'YAL034C';'YAL034W-
A';'YAL035W';'YAL036C';'YAL038W';'YAL039C';'YAL040C';'YAL041W';'YAL042W';'YAL043C';'YAL044
C';'YAL046C';'YAL047C';'YAL048C';'YAL049C';'YAL051W';'YAL053W';'YAL054C';'YAL055W';'YAL056
W';'YAL058W';'YAL059W';'YAL060W';'YAL062W';'YAL063C';'YAL064W';'YAL067C';'YAL068C';'YAR002
C-
A';'YAR002W';'YAR003W';'YAR007C';'YAR008W';'YAR014C';'YAR015W';'YAR018C';'YAR019C';'YAR020
C';'YAR027W';'YAR031W';'YAR033W';'YAR035W';'YAR042W';'YAR050W';'YAR071W';'YBL001C';'YBL002
W';'YBL003C';'YBL004W';'YBL005W';'YBL006C';'YBL007C';'YBL008W';'YBL009W';'YBL011W';'YBL013
W';'YBL014C';'YBL015W';'YBL016W';'YBL017C';'YBL018C';'YBL019W';'YBL020W';'YBL021C';'YBL022
C';'YBL023C';'YBL024W';'YBL025W';'YBL026W';'YBL027W';'YBL028C';'YBL030C';'YBL031W';'YBL032
W';'YBL033C';'YBL034C';'YBL035C';'YBL036C';'YBL037W';'YBL038W';'YBL039C';'YBL040C';'YBL041
W';'YBL042C';'YBL043W';'YBL045C';'YBL046W';'YBL047C';'YBL049W';'YBL050W';'YBL051C';'YBL052
C';'YBL054W';'YBL055C';'YBL056W';'YBL057C';'YBL058W';'YBL059C-
A';'YBL060W';'YBL061C';'YBL063W';'YBL064C';'YBL066C';'YBL067C';'YBL068W';'YBL069W';'YBL071
W-
A';'YBL072C';'YBL074C';'YBL075C';'YBL076C';'YBL078C';'YBL079W';'YBL080C';'YBL082C';'YBL084
C';'YBL085W';'YBL087C';'YBL088C';'YBL089W';'YBL090W';'YBL091C';'YBL091C-
```

```
A';'YBL092W';'YBL093C';'YBL097W';'YBL098W';'YBL099W';'YBL101C';'YBL102W';'YBL103C';'YBL104
C';'YBL105C';'YBL106C';'YBL107C';'YBL108C-
A';'YBR001C';'YBR002C';'YBR003W';'YBR004C';'YBR005W';'YBR006W';'YBR008C';'YBR009C';'YBR010
W';'YBR011C';'YBR014C';'YBR015C';'YBR016W';'YBR017C';'YBR018C';'YBR019C';'YBR020W';'YBR021
W';'YBR022W';'YBR023C';'YBR024W';'YBR025C';'YBR026C';'YBR028C';'YBR029C';'YBR030W';'YBR031
W';'YBR034C';'YBR035C';'YBR036C';'YBR037C';'YBR038W';'YBR039W';'YBR040W';'YBR041W';'YBR042
C';'YBR043C';'YBR044C';'YBR045C';'YBR046C';'YBR048W';'YBR049C';'YBR050C';'YBR052C';'YBR054
W';'YBR055C';'YBR056W';'YBR057C';'YBR058C';'YBR058C-
A';'YBR059C';'YBR060C';'YBR061C';'YBR065C';'YBR066C';'YBR067C';'YBR068C';'YBR069C';'YBR070
C';'YBR071W';'YBR072W';'YBR073W';'YBR076W';'YBR077C';'YBR078W';'YBR079C';'YBR080C';'YBR081
C';'YBR082C';'YBR083W';'YBR084C-
A';'YBR084W';'YBR085W';'YBR086C';'YBR087W';'YBR088C';'YBR089C-
A';'YBR091C';'YBR092C';'YBR093C';'YBR094W';'YBR095C';'YBR097W';'YBR098W';'YBR101C';'YBR102
C';'YBR103W';'YBR104W';'YBR105C';'YBR106W';'YBR107C';'YBR108W';'YBR109C';'YBR110W';'YBR111
C';'YBR111W-
A';'YBR112C';'YBR114W';'YBR115C';'YBR117C';'YBR118W';'YBR119W';'YBR120C';'YBR121C';'YBR122
C';'YBR123C';'YBR125C';'YBR126C';'YBR127C';'YBR128C';'YBR129C';'YBR130C';'YBR131W';'YBR132
C';'YBR133C';'YBR135W';'YBR136W';'YBR137W';'YBR139W';'YBR140C';'YBR142W';'YBR143C';'YBR145
W';'YBR146W';'YBR147W';'YBR148W';'YBR149W';'YBR150C';'YBR151W';'YBR152W';'YBR153W';'YBR154
C';'YBR155W';'YBR156C';'YBR157C';'YBR158W';'YBR159W';'YBR160W';'YBR161W';'YBR162C';'YBR162
W-
A';'YBR163W';'YBR164C';'YBR165W';'YBR166C';'YBR167C';'YBR168W';'YBR169C';'YBR170C';'YBR171
W';'YBR172C';'YBR173C';'YBR175W';'YBR176W';'YBR177C';'YBR179C';'YBR180W';'YBR181C';'YBR182
C';'YBR183W';'YBR185C';'YBR186W';'YBR188C';'YBR189W';'YBR191W';'YBR192W';'YBR193C';'YBR194
W';'YBR195C';'YBR196C';'YBR198C';'YBR199W';'YBR200W';'YBR201W';'YBR202W';'YBR203W';'YBR204
C';'YBR205W';'YBR207W';'YBR208C';'YBR210W';'YBR211C';'YBR212W';'YBR213W';'YBR214W';'YBR215
W';'YBR216C';'YBR217W';'YBR218C';'YBR221C';'YBR222C';'YBR223C';'YBR227C';'YBR228W';'YBR229
C';'YBR230C';'YBR231C';'YBR233W';'YBR233W-
A';'YBR234C';'YBR235W';'YBR236C';'YBR237W';'YBR238C';'YBR240C';'YBR243C';'YBR244W';'YBR245
C';'YBR246W';'YBR247C';'YBR248C';'YBR249C';'YBR250W';'YBR251W';'YBR252W';'YBR253W';'YBR254
C';'YBR255W';'YBR256C';'YBR257W';'YBR258C';'YBR260C';'YBR261C';'YBR262C';'YBR263W';'YBR264
C';'YBR265W';'YBR267W';'YBR268W';'YBR271W';'YBR272C';'YBR273C';'YBR274W';'YBR275C';'YBR276
C';'YBR278W';'YBR279W';'YBR280C';'YBR281C';'YBR282W';'YBR283C';'YBR286W';'YBR288C';'YBR289
W';'YBR290W';'YBR291C';'YBR293W';'YBR294W';'YBR295W';'YBR296C';'YBR297W';'YBR298C';'YBR299
W';'YBR301W';'YBR302C';'YCL001W';'YCL004W';'YCL005W';'YCL005W-
A';'YCL008C';'YCL009C';'YCL010C';'YCL011C';'YCL012C';'YCL014W';'YCL016C';'YCL017C';'YCL018
W';'YCL024W';'YCL025C';'YCL026C-
A';'YCL027W';'YCL028W';'YCL029C';'YCL030C';'YCL031C';'YCL032W';'YCL033C';'YCL034W';'YCL035
C';'YCL036W';'YCL037C';'YCL038C';'YCL039W';'YCL040W';'YCL043C';'YCL044C';'YCL045C';'YCL047
C';'YCL048W';'YCL050C';'YCL051W';'YCL052C';'YCL054W';'YCL055W';'YCL056C';'YCL057C-
A';'YCL057W';'YCL058C';'YCL058W-
A';'YCL059C';'YCL061C';'YCL063W';'YCL064C';'YCL066W';'YCL067C';'YCL069W';'YCL073C';'YCR002
C';'YCR003W';'YCR004C';'YCR005C';'YCR008W';'YCR009C';'YCR010C';'YCR011C';'YCR012W';'YCR014
C';'YCR017C';'YCR018C';'YCR019W';'YCR020C';'YCR020C-A';'YCR020W-
B';'YCR021C';'YCR023C';'YCR024C';'YCR024C-A';'YCR026C';'YCR027C';'YCR028C';'YCR028C-
A';'YCR030C';'YCR031C';'YCR032W';'YCR033W';'YCR034W';'YCR035C';'YCR036W';'YCR037C';'YCR038
C';'YCR039C';'YCR040W';'YCR042C';'YCR044C';'YCR045C';'YCR046C';'YCR047C';'YCR048W';'YCR052
W';'YCR053W';'YCR054C';'YCR057C';'YCR059C';'YCR060W';'YCR063W';'YCR065W';'YCR066W';'YCR067
C';'YCR068W';'YCR069W';'YCR071C';'YCR072C';'YCR073C';'YCR073W-
A';'YCR075C';'YCR076C';'YCR077C';'YCR079W';'YCR081W';'YCR082W';'YCR083W';'YCR084C';'YCR086
W';'YCR088W';'YCR089W';'YCR091W';'YCR092C';'YCR093W';'YCR094W';'YCR096C';'YCR097W';'YCR098
C';'YCR104W';'YCR105W';'YCR106W';'YCR107W';'YDL001W';'YDL002C';'YDL003W';'YDL004W';'YDL005
C';'YDL006W';'YDL007W';'YDL008W';'YDL010W';'YDL012C';'YDL013W';'YDL014W';'YDL015C';'YDL017
W';'YDL018C';'YDL019C';'YDL020C';'YDL021W';'YDL022W';'YDL024C';'YDL025C';'YDL028C';'YDL029
W';'YDL030W';'YDL031W';'YDL033C';'YDL035C';'YDL036C';'YDL037C';'YDL039C';'YDL040C';'YDL042
C';'YDL043C';'YDL044C';'YDL045C';'YDL045W-
A';'YDL046W';'YDL047W';'YDL048C';'YDL049C';'YDL051W';'YDL052C';'YDL053C';'YDL054C';'YDL055
C';'YDL056W';'YDL058W';'YDL059C';'YDL060W';'YDL061C';'YDL063C';'YDL064W';'YDL065C';'YDL066
W';'YDL067C';'YDL069C';'YDL070W';'YDL072C';'YDL074C';'YDL075W';'YDL076C';'YDL077C';'YDL078
C';'YDL079C';'YDL080C';'YDL081C';'YDL082W';'YDL083C';'YDL084W';'YDL085W';'YDL087C';'YDL088
```

```
C';'YDL089W';'YDL090C';'YDL091C';'YDL092W';'YDL093W';'YDL095W';'YDL097C';'YDL098C';'YDL099
W';'YDL100C';'YDL101C';'YDL102W';'YDL103C';'YDL104C';'YDL105W';'YDL106C';'YDL107W';'YDL108
W';'YDL110C';'YDL111C';'YDL112W';'YDL113C';'YDL115C';'YDL116W';'YDL117W';'YDL120W';'YDL122
W';'YDL123W';'YDL124W';'YDL125C';'YDL126C';'YDL127W';'YDL128W';'YDL130W';'YDL130W-
A';'YDL131W';'YDL132W';'YDL133C-
A';'YDL133W';'YDL134C';'YDL135C';'YDL136W';'YDL137W';'YDL138W';'YDL139C';'YDL140C';'YDL141
W';'YDL142C';'YDL143W';'YDL145C';'YDL146W';'YDL147W';'YDL148C';'YDL149W';'YDL150W';'YDL153
C';'YDL154W';'YDL155W';'YDL156W';'YDL159W';'YDL160C';'YDL160C-
A';'YDL161W';'YDL164C';'YDL165W';'YDL166C';'YDL167C';'YDL168W';'YDL169C';'YDL170W';'YDL171
C';'YDL173W';'YDL174C';'YDL175C';'YDL176W';'YDL178W';'YDL179W';'YDL181W';'YDL182W';'YDL183
C';'YDL184C';'YDL185W';'YDL188C';'YDL189W';'YDL190C';'YDL191W';'YDL192W';'YDL193W';'YDL194
W';'YDL195W';'YDL197C';'YDL198C';'YDL200C';'YDL201W';'YDL202W';'YDL203C';'YDL204W';'YDL205
C';'YDL207W';'YDL208W';'YDL209C';'YDL210W';'YDL212W';'YDL213C';'YDL214C';'YDL215C';'YDL216
C';'YDL217C';'YDL219W';'YDL220C';'YDL222C';'YDL223C';'YDL224C';'YDL225W';'YDL226C';'YDL227
C';'YDL229W';'YDL230W';'YDL231C';'YDL232W';'YDL234C';'YDL235C';'YDL236W';'YDL237W';'YDL238
C';'YDL239C';'YDL240W';'YDL243C';'YDL244W';'YDL245C';'YDL247W';'YDL248W';'YDR001C';'YDR002
W';'YDR003W';'YDR004W';'YDR005C';'YDR006C';'YDR007W';'YDR009W';'YDR011W';'YDR012W';'YDR013
W';'YDR014W';'YDR014W-
A';'YDR016C';'YDR017C';'YDR019C';'YDR021W';'YDR022C';'YDR023W';'YDR025W';'YDR026C';'YDR027
C';'YDR028C';'YDR030C';'YDR031W';'YDR032C';'YDR033W';'YDR034C';'YDR035W';'YDR036C';'YDR037
W';'YDR038C';'YDR039C';'YDR040C';'YDR041W';'YDR043C';'YDR044W';'YDR045C';'YDR046C';'YDR047
W';'YDR049W';'YDR050C';'YDR051C';'YDR052C';'YDR054C';'YDR055W';'YDR057W';'YDR058C';'YDR059
C';'YDR060W';'YDR062W';'YDR063W';'YDR064W';'YDR065W';'YDR068W';'YDR069C';'YDR071C';'YDR072
C';'YDR073W';'YDR074W';'YDR075W';'YDR076W';'YDR077W';'YDR078C';'YDR079C-
A';'YDR079W';'YDR080W';'YDR081C';'YDR082W';'YDR083W';'YDR084C';'YDR085C';'YDR086C';'YDR087
C';'YDR088C';'YDR091C';'YDR092W';'YDR093W';'YDR096W';'YDR097C';'YDR098C';'YDR099W';'YDR100
W';'YDR101C';'YDR103W';'YDR104C';'YDR105C';'YDR106W';'YDR107C';'YDR108W';'YDR110W';'YDR113
C';'YDR116C';'YDR117C';'YDR118W';'YDR119W-
A';'YDR120C';'YDR121W';'YDR122W';'YDR123C';'YDR125C';'YDR126W';'YDR127W';'YDR128W';'YDR129
C';'YDR130C';'YDR135C';'YDR137W';'YDR138W';'YDR139C';'YDR140W';'YDR141C';'YDR142C';'YDR143
C';'YDR144C';'YDR145W';'YDR146C';'YDR147W';'YDR148C';'YDR150W';'YDR151C';'YDR152W';'YDR153
C';'YDR155C';'YDR156W';'YDR158W';'YDR159W';'YDR160W';'YDR162C';'YDR163W';'YDR164C';'YDR165
W';'YDR166C';'YDR167W';'YDR168W';'YDR169C';'YDR170C';'YDR171W';'YDR172W';'YDR173C';'YDR174
W';'YDR175C';'YDR176W';'YDR177W';'YDR178W';'YDR179C';'YDR180W';'YDR181C';'YDR182W';'YDR183
W';'YDR184C';'YDR185C';'YDR186C';'YDR188W';'YDR189W';'YDR190C';'YDR191W';'YDR192C';'YDR194
C';'YDR195W';'YDR196C';'YDR197W';'YDR198C';'YDR200C';'YDR201W';'YDR202C';'YDR204W';'YDR205
W';'YDR206W';'YDR207C';'YDR208W';'YDR211W';'YDR212W';'YDR213W';'YDR214W';'YDR216W';'YDR217
C';'YDR218C';'YDR219C';'YDR221W';'YDR223W';'YDR224C';'YDR225W';'YDR226W';'YDR227W';'YDR228
C';'YDR229W';'YDR231C';'YDR232W';'YDR233C';'YDR234W';'YDR235W';'YDR236C';'YDR237W';'YDR238
C';'YDR239C';'YDR240C';'YDR242W';'YDR243C';'YDR244W';'YDR245W';'YDR246W';'YDR247W';'YDR251
W';'YDR252W';'YDR253C';'YDR254W';'YDR255C';'YDR256C';'YDR257C';'YDR258C';'YDR259C';'YDR260
C';'YDR261C';'YDR263C';'YDR264C';'YDR265W';'YDR266C';'YDR267C';'YDR268W';'YDR270W';'YDR272
W';'YDR273W';'YDR275W';'YDR276C';'YDR277C';'YDR279W';'YDR280W';'YDR281C';'YDR283C';'YDR284
C';'YDR285W';'YDR287W';'YDR288W';'YDR289C';'YDR291W';'YDR292C';'YDR293C';'YDR294C';'YDR295
C';'YDR296W';'YDR297W';'YDR298C';'YDR299W';'YDR300C';'YDR301W';'YDR302W';'YDR303C';'YDR304
C';'YDR305C';'YDR308C';'YDR309C';'YDR310C';'YDR311W';'YDR312W';'YDR313C';'YDR314C';'YDR315
C';'YDR316W';'YDR317W';'YDR318W';'YDR320C';'YDR320C-A';'YDR321W';'YDR322C-
A';'YDR322W';'YDR323C';'YDR324C';'YDR325W';'YDR326C';'YDR328C';'YDR329C';'YDR330W';'YDR331
W';'YDR332W';'YDR333C';'YDR334W';'YDR335W';'YDR337W';'YDR339C';'YDR341C';'YDR342C';'YDR343
C';'YDR345C';'YDR346C';'YDR347W';'YDR348C';'YDR349C';'YDR350C';'YDR351W';'YDR352W';'YDR353
W';'YDR354W';'YDR356W';'YDR358W';'YDR359C';'YDR361C';'YDR362C';'YDR363W';'YDR363W-
A';'YDR364C';'YDR365C';'YDR367W';'YDR368W';'YDR369C';'YDR372C';'YDR373W';'YDR374W-
A';'YDR375C';'YDR376W';'YDR377W';'YDR378C';'YDR379C-A';'YDR379W';'YDR380W';'YDR381C-
A';'YDR381W';'YDR382W';'YDR383C';'YDR384C';'YDR385W';'YDR386W';'YDR388W';'YDR389W';'YDR390
C';'YDR392W';'YDR393W';'YDR394W';'YDR395W';'YDR397C';'YDR398W';'YDR399W';'YDR400W';'YDR402
C';'YDR403W';'YDR404C';'YDR405W';'YDR406W';'YDR407C';'YDR408C';'YDR409W';'YDR410C';'YDR411
C';'YDR412W';'YDR414C';'YDR416W';'YDR418W';'YDR419W';'YDR420W';'YDR421W';'YDR422C';'YDR423
C';'YDR424C';'YDR425W';'YDR427W';'YDR428C';'YDR429C';'YDR430C';'YDR432W';'YDR434W';'YDR435
C';'YDR436W';'YDR437W';'YDR438W';'YDR439W';'YDR440W';'YDR441C';'YDR443C';'YDR446W';'YDR447
C';'YDR448W';'YDR449C';'YDR450W';'YDR451C';'YDR452W';'YDR453C';'YDR454C';'YDR456W';'YDR457
```

```
W';'YDR458C';'YDR459C';'YDR460W';'YDR461W';'YDR462W';'YDR463W';'YDR464W';'YDR465C';'YDR466
W';'YDR468C';'YDR469W';'YDR470C';'YDR471W';'YDR472W';'YDR473C';'YDR475C';'YDR477W';'YDR478
W';'YDR479C';'YDR480W';'YDR481C';'YDR482C';'YDR483W';'YDR484W';'YDR485C';'YDR486C';'YDR487
C';'YDR488C';'YDR489W';'YDR490C';'YDR492W';'YDR493W';'YDR494W';'YDR495C';'YDR496C';'YDR497
C';'YDR498C';'YDR499W';'YDR500C';'YDR501W';'YDR502C';'YDR503C';'YDR504C';'YDR505C';'YDR506
C';'YDR507C';'YDR508C';'YDR510W';'YDR511W';'YDR512C';'YDR513W';'YDR514C';'YDR515W';'YDR516
C';'YDR517W';'YDR518W';'YDR519W';'YDR522C';'YDR523C';'YDR524C';'YDR525W-
A';'YDR527W';'YDR528W';'YDR529C';'YDR530C';'YDR531W';'YDR532C';'YDR533C';'YDR534C';'YDR536
W';'YDR538W';'YDR539W';'YDR540C';'YDR542W';'YDR545W';'YEL001C';'YEL002C';'YEL003W';'YEL004
W';'YEL005C';'YEL006W';'YEL007W';'YEL009C';'YEL011W';'YEL012W';'YEL013W';'YEL015W';'YEL016
C';'YEL017C-A';'YEL017W';'YEL018W';'YEL019C';'YEL020W-
A';'YEL021W';'YEL022W';'YEL024W';'YEL026W';'YEL027W';'YEL029C';'YEL030W';'YEL031W';'YEL032
W';'YEL034W';'YEL036C';'YEL037C';'YEL038W';'YEL039C';'YEL040W';'YEL041W';'YEL042W';'YEL043
W';'YEL044W';'YEL046C';'YEL047C';'YEL048C';'YEL049W';'YEL050C';'YEL051W';'YEL052W';'YEL053
C';'YEL054C';'YEL055C';'YEL056W';'YEL058W';'YEL059C-
A';'YEL060C';'YEL061C';'YEL062W';'YEL063C';'YEL064C';'YEL065W';'YEL066W';'YEL069C';'YEL071
W';'YEL072W';'YER001W';'YER002W';'YER003C';'YER004W';'YER005W';'YER006W';'YER007C-
A';'YER007W';'YER008C';'YER009W';'YER010C';'YER011W';'YER012W';'YER013W';'YER014C-
A';'YER014W';'YER015W';'YER016W';'YER017C';'YER018C';'YER019C-
A';'YER019W';'YER020W';'YER021W';'YER022W';'YER023W';'YER024W';'YER025W';'YER026C';'YER027
C';'YER028C';'YER029C';'YER030W';'YER031C';'YER032W';'YER033C';'YER035W';'YER036C';'YER037
W';'YER038C';'YER039C';'YER040W';'YER041W';'YER042W';'YER043C';'YER044C';'YER044C-
A';'YER045C';'YER046W';'YER047C';'YER048C';'YER048W-
A';'YER049W';'YER050C';'YER051W';'YER052C';'YER053C';'YER054C';'YER055C';'YER056C';'YER056
C-A';'YER057C';'YER058W';'YER059W';'YER060W';'YER060W-
A';'YER061C';'YER062C';'YER063W';'YER065C';'YER067W';'YER068W';'YER069W';'YER070W';'YER072
W';'YER073W';'YER074W';'YER074W-
A';'YER075C';'YER078C';'YER080W';'YER081W';'YER082C';'YER083C';'YER086W';'YER087C-
B';'YER087W';'YER088C';'YER089C';'YER090W';'YER091C';'YER092W';'YER093C';'YER093C-
A';'YER094C';'YER095W';'YER096W';'YER098W';'YER099C';'YER100W';'YER101C';'YER102W';'YER103
W';'YER104W';'YER105C';'YER106W';'YER107C';'YER109C';'YER110C';'YER111C';'YER112W';'YER113
C';'YER114C';'YER115C';'YER116C';'YER117W';'YER118C';'YER119C';'YER120W';'YER122C';'YER123
W';'YER124C';'YER125W';'YER126C';'YER127W';'YER128W';'YER129W';'YER131W';'YER132C';'YER133
W';'YER134C';'YER136W';'YER139C';'YER140W';'YER141W';'YER142C';'YER143W';'YER144C';'YER145
C';'YER146W';'YER147C';'YER148W';'YER149C';'YER150W';'YER151C';'YER152C';'YER153C';'YER154
W';'YER155C';'YER157W';'YER159C';'YER161C';'YER162C';'YER163C';'YER164W';'YER165W';'YER166
W';'YER167W';'YER168C';'YER169W';'YER170W';'YER171W';'YER172C';'YER173W';'YER174C';'YER175
C';'YER176W';'YER177W';'YER178W';'YER179W';'YER180C';'YER180C-
A';'YER183C';'YER185W';'YER190W';'YFL001W';'YFL002C';'YFL003C';'YFL004W';'YFL005W';'YFL007
W';'YFL008W';'YFL009W';'YFL010C';'YFL010W-
A';'YFL011W';'YFL013C';'YFL014W';'YFL016C';'YFL017C';'YFL017W-
A';'YFL018C';'YFL020C';'YFL021W';'YFL022C';'YFL023W';'YFL024C';'YFL025C';'YFL026W';'YFL027
C';'YFL028C';'YFL029C';'YFL030W';'YFL031W';'YFL033C';'YFL034C-A';'YFL034C-
B';'YFL036W';'YFL037W';'YFL038C';'YFL039C';'YFL041W';'YFL044C';'YFL045C';'YFL047W';'YFL048
C';'YFL049W';'YFL050C';'YFL053W';'YFL055W';'YFL056C';'YFL057C';'YFL058W';'YFL059W';'YFL060
C';'YFL062W';'YFR001W';'YFR002W';'YFR003C';'YFR004W';'YFR005C';'YFR007W';'YFR008W';'YFR009
W';'YFR010W';'YFR011C';'YFR012W';'YFR013W';'YFR014C';'YFR015C';'YFR016C';'YFR017C';'YFR019
W';'YFR021W';'YFR022W';'YFR023W';'YFR024C-
A';'YFR025C';'YFR026C';'YFR027W';'YFR028C';'YFR029W';'YFR030W';'YFR031C';'YFR031C-
A';'YFR032C-
A';'YFR033C';'YFR034C';'YFR036W';'YFR037C';'YFR038W';'YFR040W';'YFR041C';'YFR042W';'YFR043
C';'YFR044C';'YFR046C';'YFR047C';'YFR048W';'YFR049W';'YFR050C';'YFR051C';'YFR052W';'YFR053
C';'YGL001C';'YGL002W';'YGL003C';'YGL004C';'YGL005C';'YGL006W';'YGL008C';'YGL009C';'YGL011
C';'YGL012W';'YGL013C';'YGL014W';'YGL016W';'YGL017W';'YGL018C';'YGL019W';'YGL020C';'YGL021
W';'YGL022W';'YGL023C';'YGL025C';'YGL026C';'YGL027C';'YGL028C';'YGL029W';'YGL030W';'YGL031
C';'YGL032C';'YGL033W';'YGL035C';'YGL037C';'YGL038C';'YGL039W';'YGL040C';'YGL043W';'YGL044
C';'YGL045W';'YGL047W';'YGL048C';'YGL049C';'YGL050W';'YGL051W';'YGL053W';'YGL054C';'YGL055
W';'YGL056C';'YGL057C';'YGL058W';'YGL059W';'YGL060W';'YGL061C';'YGL062W';'YGL063W';'YGL064
C';'YGL065C';'YGL066W';'YGL067W';'YGL068W';'YGL070C';'YGL071W';'YGL073W';'YGL075C';'YGL076
C';'YGL077C';'YGL078C';'YGL080W';'YGL083W';'YGL084C';'YGL086W';'YGL087C';'YGL089C';'YGL090
```

```
W';'YGL091C';'YGL092W';'YGL093W';'YGL094C';'YGL095C';'YGL096W';'YGL097W';'YGL098W';'YGL099↙
W';'YGL100W';'YGL103W';'YGL104C';'YGL105W';'YGL106W';'YGL107C';'YGL110C';'YGL111W';'YGL112↙
C';'YGL113W';'YGL115W';'YGL116W';'YGL119W';'YGL120C';'YGL121C';'YGL122C';'YGL123W';'YGL124↙
C';'YGL125W';'YGL126W';'YGL127C';'YGL128C';'YGL129C';'YGL130W';'YGL131C';'YGL133W';'YGL134↙
W';'YGL135W';'YGL136C';'YGL137W';'YGL139W';'YGL141W';'YGL142C';'YGL143C';'YGL144C';'YGL145↙
W';'YGL147C';'YGL148W';'YGL150C';'YGL151W';'YGL153W';'YGL154C';'YGL155W';'YGL156W';'YGL157↙
W';'YGL158W';'YGL160W';'YGL161C';'YGL162W';'YGL163C';'YGL164C';'YGL166W';'YGL167C';'YGL168↙
W';'YGL169W';'YGL170C';'YGL171W';'YGL172W';'YGL173C';'YGL174W';'YGL175C';'YGL178W';'YGL179↙
C';'YGL180W';'YGL181W';'YGL183C';'YGL184C';'YGL186C';'YGL187C';'YGL189C';'YGL190C';'YGL191↙
W';'YGL192W';'YGL194C';'YGL195W';'YGL196W';'YGL197W';'YGL198W';'YGL200C';'YGL201C';'YGL202↙
W';'YGL203C';'YGL205W';'YGL206C';'YGL207W';'YGL208W';'YGL209W';'YGL210W';'YGL211W';'YGL212↙
W';'YGL213C';'YGL215W';'YGL216W';'YGL219C';'YGL220W';'YGL221C';'YGL222C';'YGL223C';'YGL224↙
C';'YGL225W';'YGL226C-↙
A';'YGL226W';'YGL227W';'YGL228W';'YGL229C';'YGL231C';'YGL232W';'YGL233W';'YGL234W';'YGL236↙
C';'YGL237C';'YGL238W';'YGL240W';'YGL241W';'YGL243W';'YGL244W';'YGL245W';'YGL246C';'YGL247↙
W';'YGL248W';'YGL249W';'YGL250W';'YGL251C';'YGL252C';'YGL253W';'YGL254W';'YGL255W';'YGL256↙
W';'YGL257C';'YGL258W';'YGL263W';'YGR002C';'YGR003W';'YGR004W';'YGR005C';'YGR006W';'YGR007↙
W';'YGR008C';'YGR009C';'YGR010W';'YGR012W';'YGR013W';'YGR014W';'YGR019W';'YGR020C';'YGR023↙
W';'YGR024C';'YGR027C';'YGR028W';'YGR029W';'YGR030C';'YGR031C-↙
A';'YGR031W';'YGR032W';'YGR033C';'YGR034W';'YGR036C';'YGR037C';'YGR038W';'YGR040W';'YGR041↙
W';'YGR043C';'YGR044C';'YGR046W';'YGR047C';'YGR048W';'YGR049W';'YGR054W';'YGR055W';'YGR056↙
W';'YGR057C';'YGR058W';'YGR059W';'YGR060W';'YGR061C';'YGR062C';'YGR063C';'YGR065C';'YGR068↙
C';'YGR070W';'YGR071C';'YGR072W';'YGR074W';'YGR075C';'YGR076C';'YGR077C';'YGR078C';'YGR080↙
W';'YGR081C';'YGR082W';'YGR083C';'YGR084C';'YGR085C';'YGR086C';'YGR087C';'YGR088W';'YGR089↙
W';'YGR090W';'YGR091W';'YGR092W';'YGR094W';'YGR095C';'YGR096W';'YGR097W';'YGR098C';'YGR099↙
W';'YGR100W';'YGR101W';'YGR102C';'YGR103W';'YGR104C';'YGR105W';'YGR106C';'YGR108W';'YGR109↙
C';'YGR110W';'YGR112W';'YGR113W';'YGR116W';'YGR118W';'YGR119C';'YGR120C';'YGR121C';'YGR122↙
W';'YGR123C';'YGR124W';'YGR128C';'YGR129W';'YGR130C';'YGR131W';'YGR132C';'YGR133W';'YGR134↙
W';'YGR135W';'YGR136W';'YGR138C';'YGR140W';'YGR141W';'YGR142W';'YGR143W';'YGR144W';'YGR145↙
W';'YGR146C';'YGR147C';'YGR148C';'YGR150C';'YGR152C';'YGR154C';'YGR155W';'YGR156W';'YGR157↙
W';'YGR158C';'YGR159C';'YGR162W';'YGR163W';'YGR165W';'YGR166W';'YGR167W';'YGR169C';'YGR170↙
W';'YGR171C';'YGR172C';'YGR173W';'YGR174C';'YGR175C';'YGR177C';'YGR178C';'YGR179C';'YGR180↙
C';'YGR181W';'YGR183C';'YGR184C';'YGR185C';'YGR186W';'YGR187C';'YGR188C';'YGR189C';'YGR191↙
W';'YGR192C';'YGR193C';'YGR194C';'YGR195W';'YGR196C';'YGR197C';'YGR198W';'YGR199W';'YGR200↙
C';'YGR202C';'YGR203W';'YGR204W';'YGR205W';'YGR206W';'YGR207C';'YGR208W';'YGR209C';'YGR211↙
W';'YGR212W';'YGR213C';'YGR214W';'YGR215W';'YGR216C';'YGR217W';'YGR218W';'YGR220C';'YGR221↙
C';'YGR222W';'YGR223C';'YGR224W';'YGR225W';'YGR227W';'YGR229C';'YGR230W';'YGR231C';'YGR232↙
W';'YGR233C';'YGR234W';'YGR235C';'YGR236C';'YGR238C';'YGR239C';'YGR240C';'YGR241C';'YGR243↙
W';'YGR244C';'YGR245C';'YGR246C';'YGR247W';'YGR248W';'YGR249W';'YGR250C';'YGR251W';'YGR252↙
W';'YGR253C';'YGR254W';'YGR255C';'YGR256W';'YGR257C';'YGR258C';'YGR260W';'YGR261C';'YGR262↙
C';'YGR263C';'YGR264C';'YGR266W';'YGR267C';'YGR268C';'YGR270W';'YGR271C-↙
A';'YGR271W';'YGR274C';'YGR275W';'YGR276C';'YGR277C';'YGR278W';'YGR279C';'YGR280C';'YGR281↙
W';'YGR282C';'YGR283C';'YGR284C';'YGR285C';'YGR286C';'YGR287C';'YGR288W';'YGR289C';'YGR292↙
W';'YGR294W';'YGR295C';'YGR296W';'YHL001W';'YHL002W';'YHL003C';'YHL004W';'YHL006C';'YHL007↙
C';'YHL009C';'YHL010C';'YHL011C';'YHL013C';'YHL014C';'YHL015W';'YHL016C';'YHL019C';'YHL020↙
C';'YHL021C';'YHL022C';'YHL023C';'YHL024W';'YHL025W';'YHL027W';'YHL028W';'YHL030W';'YHL031↙
C';'YHL032C';'YHL033C';'YHL034C';'YHL035C';'YHL036W';'YHL038C';'YHL039W';'YHL040C';'YHL043↙
W';'YHL046C';'YHL047C';'YHL048W';'YHR001W';'YHR001W-↙
A';'YHR002W';'YHR003C';'YHR004C';'YHR005C';'YHR005C-↙
A';'YHR006W';'YHR007C';'YHR008C';'YHR010W';'YHR011W';'YHR012W';'YHR013C';'YHR014W';'YHR015↙
W';'YHR016C';'YHR017W';'YHR018C';'YHR019C';'YHR020W';'YHR021C';'YHR023W';'YHR024C';'YHR025↙
W';'YHR026W';'YHR027C';'YHR028C';'YHR029C';'YHR030C';'YHR031C';'YHR032W';'YHR034C';'YHR036↙
W';'YHR037W';'YHR038W';'YHR039C';'YHR039C-↙
A';'YHR040W';'YHR041C';'YHR042W';'YHR043C';'YHR044C';'YHR046C';'YHR047C';'YHR049W';'YHR050↙
W';'YHR051W';'YHR052W';'YHR053C';'YHR055C';'YHR056C';'YHR057C';'YHR058C';'YHR059W';'YHR060↙
W';'YHR061C';'YHR062C';'YHR063C';'YHR064C';'YHR065C';'YHR066W';'YHR067W';'YHR068W';'YHR069↙
C';'YHR070W';'YHR071W';'YHR072W';'YHR072W-↙
A';'YHR073W';'YHR074W';'YHR075C';'YHR076W';'YHR077C';'YHR079C';'YHR079C-↙
A';'YHR080C';'YHR081W';'YHR082C';'YHR083W';'YHR084W';'YHR085W';'YHR086W';'YHR087W';'YHR088↙
W';'YHR089C';'YHR090C';'YHR091C';'YHR092C';'YHR094C';'YHR096C';'YHR098C';'YHR099W';'YHR100↙
```

```
C';'YHR101C';'YHR102W';'YHR103W';'YHR104W';'YHR105W';'YHR106W';'YHR107C';'YHR108W';'YHR109
W';'YHR110W';'YHR111W';'YHR112C';'YHR113W';'YHR114W';'YHR115C';'YHR116W';'YHR117W';'YHR118
C';'YHR119W';'YHR120W';'YHR121W';'YHR122W';'YHR123W';'YHR124W';'YHR127W';'YHR128W';'YHR129
C';'YHR132C';'YHR132W-
A';'YHR133C';'YHR134W';'YHR135C';'YHR136C';'YHR137W';'YHR139C';'YHR141C';'YHR142W';'YHR143
W';'YHR143W-
A';'YHR144C';'YHR146W';'YHR147C';'YHR148W';'YHR149C';'YHR150W';'YHR151C';'YHR152W';'YHR153
C';'YHR154W';'YHR155W';'YHR156C';'YHR157W';'YHR158C';'YHR160C';'YHR161C';'YHR162W';'YHR163
W';'YHR164C';'YHR165C';'YHR166C';'YHR167W';'YHR168W';'YHR169W';'YHR170W';'YHR171W';'YHR172
W';'YHR174W';'YHR175W';'YHR176W';'YHR178W';'YHR179W';'YHR181W';'YHR183W';'YHR184W';'YHR185
C';'YHR186C';'YHR187W';'YHR188C';'YHR189W';'YHR190W';'YHR191C';'YHR192W';'YHR193C';'YHR194
W';'YHR195W';'YHR196W';'YHR197W';'YHR198C';'YHR199C';'YHR199C-
A';'YHR200W';'YHR201C';'YHR203C';'YHR204W';'YHR205W';'YHR206W';'YHR207C';'YHR208W';'YHR209
W';'YHR211W';'YHR215W';'YHR216W';'YIL002C';'YIL003W';'YIL004C';'YIL005W';'YIL006W';'YIL007
C';'YIL008W';'YIL009C-
A';'YIL009W';'YIL010W';'YIL011W';'YIL013C';'YIL014W';'YIL015W';'YIL016W';'YIL017C';'YIL018
W';'YIL019W';'YIL020C';'YIL021W';'YIL022W';'YIL023C';'YIL026C';'YIL027C';'YIL030C';'YIL031
W';'YIL033C';'YIL034C';'YIL035C';'YIL036W';'YIL037C';'YIL038C';'YIL039W';'YIL040W';'YIL041
W';'YIL042C';'YIL043C';'YIL044C';'YIL045W';'YIL046W';'YIL047C';'YIL048W';'YIL049W';'YIL050
W';'YIL051C';'YIL052C';'YIL053W';'YIL056W';'YIL057C';'YIL061C';'YIL062C';'YIL063C';'YIL064
W';'YIL065C';'YIL066C';'YIL068C';'YIL069C';'YIL070C';'YIL071C';'YIL072W';'YIL073C';'YIL074
C';'YIL075C';'YIL076W';'YIL078W';'YIL079C';'YIL083C';'YIL084C';'YIL085C';'YIL087C';'YIL088
C';'YIL089W';'YIL090W';'YIL091C';'YIL093C';'YIL094C';'YIL095W';'YIL097W';'YIL098C';'YIL099
W';'YIL101C';'YIL103W';'YIL104C';'YIL105C';'YIL106W';'YIL107C';'YIL109C';'YIL110W';'YIL111
W';'YIL112W';'YIL113W';'YIL114C';'YIL115C';'YIL116W';'YIL117C';'YIL118W';'YIL119C';'YIL120
W';'YIL121W';'YIL122W';'YIL123W';'YIL124W';'YIL125W';'YIL126W';'YIL128W';'YIL129C';'YIL130
W';'YIL131C';'YIL132C';'YIL133C';'YIL134W';'YIL135C';'YIL136W';'YIL137C';'YIL138C';'YIL139
C';'YIL140W';'YIL142W';'YIL143C';'YIL144W';'YIL145C';'YIL146C';'YIL147C';'YIL148W';'YIL149
C';'YIL150C';'YIL153W';'YIL154C';'YIL155C';'YIL156W';'YIL157C';'YIL158W';'YIL159W';'YIL160
C';'YIL162W';'YIL164C';'YIL172C';'YIL173W';'YIR001C';'YIR002C';'YIR003W';'YIR004W';'YIR005
W';'YIR006C';'YIR008C';'YIR009W';'YIR010W';'YIR011C';'YIR012W';'YIR013C';'YIR015W';'YIR017
C';'YIR018W';'YIR019C';'YIR021W';'YIR022W';'YIR023W';'YIR024C';'YIR025W';'YIR026C';'YIR027
C';'YIR028W';'YIR029W';'YIR030C';'YIR031C';'YIR032C';'YIR033W';'YIR034C';'YIR037W';'YIR038
C';'YIR039C';'YIR041W';'YJL001W';'YJL002C';'YJL003W';'YJL004C';'YJL005W';'YJL006C';'YJL008
C';'YJL010C';'YJL011C';'YJL012C';'YJL013C';'YJL014W';'YJL019W';'YJL020C';'YJL023C';'YJL024
C';'YJL025W';'YJL026W';'YJL028W';'YJL029C';'YJL030W';'YJL031C';'YJL033W';'YJL034W';'YJL035
C';'YJL036W';'YJL037W';'YJL038C';'YJL039C';'YJL041W';'YJL042W';'YJL044C';'YJL045W';'YJL046
W';'YJL047C';'YJL048C';'YJL050W';'YJL051W';'YJL052W';'YJL053W';'YJL054W';'YJL056C';'YJL057
C';'YJL058C';'YJL059W';'YJL060W';'YJL061W';'YJL062W';'YJL062W-
A';'YJL063C';'YJL065C';'YJL066C';'YJL068C';'YJL069C';'YJL071W';'YJL072C';'YJL073W';'YJL074
C';'YJL076W';'YJL077C';'YJL078C';'YJL079C';'YJL080C';'YJL081C';'YJL082W';'YJL083W';'YJL084
C';'YJL085W';'YJL087C';'YJL088W';'YJL089W';'YJL090C';'YJL091C';'YJL092W';'YJL093C';'YJL094
C';'YJL095W';'YJL096W';'YJL097W';'YJL098W';'YJL099W';'YJL100W';'YJL101C';'YJL102W';'YJL103
C';'YJL104W';'YJL105W';'YJL106W';'YJL108C';'YJL109C';'YJL110C';'YJL111W';'YJL112W';'YJL115
W';'YJL116C';'YJL117W';'YJL118W';'YJL121C';'YJL122W';'YJL123C';'YJL124C';'YJL125C';'YJL126
W';'YJL127C';'YJL128C';'YJL129C';'YJL130C';'YJL131C';'YJL133W';'YJL134W';'YJL136C';'YJL137
C';'YJL138C';'YJL139C';'YJL140W';'YJL141C';'YJL143W';'YJL144W';'YJL145W';'YJL146W';'YJL148
W';'YJL149W';'YJL151C';'YJL153C';'YJL154C';'YJL155C';'YJL156C';'YJL157C';'YJL158C';'YJL159
W';'YJL162C';'YJL164C';'YJL165C';'YJL166W';'YJL167W';'YJL168C';'YJL170C';'YJL171C';'YJL172
W';'YJL173C';'YJL174W';'YJL176C';'YJL177W';'YJL178C';'YJL179W';'YJL180C';'YJL183W';'YJL184
W';'YJL185C';'YJL186W';'YJL187C';'YJL189W';'YJL190C';'YJL191W';'YJL192C';'YJL194W';'YJL196
C';'YJL197W';'YJL198W';'YJL200C';'YJL201W';'YJL203W';'YJL204C';'YJL205C';'YJL207C';'YJL208
C';'YJL209W';'YJL210W';'YJL212C';'YJL213W';'YJL214W';'YJL216C';'YJL217W';'YJL219W';'YJL221
C';'YJL222W';'YJL223C';'YJR001W';'YJR002W';'YJR004C';'YJR005W';'YJR006W';'YJR007W';'YJR008
W';'YJR009C';'YJR010C-
A';'YJR010W';'YJR013W';'YJR014W';'YJR016C';'YJR017C';'YJR019C';'YJR021C';'YJR022W';'YJR024
C';'YJR025C';'YJR031C';'YJR032W';'YJR033C';'YJR034W';'YJR035W';'YJR036C';'YJR040W';'YJR041
C';'YJR042W';'YJR043C';'YJR044C';'YJR045C';'YJR046W';'YJR047C';'YJR048W';'YJR049C';'YJR050
W';'YJR051W';'YJR052W';'YJR053W';'YJR054W';'YJR055W';'YJR057W';'YJR058C';'YJR059W';'YJR060
W';'YJR062C';'YJR063W';'YJR064W';'YJR065C';'YJR066W';'YJR067C';'YJR068W';'YJR069C';'YJR070
```

```
C';'YJR072C';'YJR073C';'YJR074W';'YJR075W';'YJR076C';'YJR077C';'YJR078W';'YJR080C';'YJR082↙
C';'YJR083C';'YJR084W';'YJR086W';'YJR088C';'YJR089W';'YJR090C';'YJR091C';'YJR092W';'YJR093↙
C';'YJR094C';'YJR094W-↙
A';'YJR095W';'YJR096W';'YJR097W';'YJR099W';'YJR100C';'YJR101W';'YJR102C';'YJR103W';'YJR104↙
C';'YJR105W';'YJR106W';'YJR108W';'YJR109C';'YJR110W';'YJR112W';'YJR113C';'YJR117W';'YJR118↙
C';'YJR119C';'YJR120W';'YJR121W';'YJR122W';'YJR123W';'YJR125C';'YJR126C';'YJR127C';'YJR130↙
C';'YJR131W';'YJR132W';'YJR133W';'YJR134C';'YJR135C';'YJR135W-↙
A';'YJR136C';'YJR137C';'YJR138W';'YJR139C';'YJR140C';'YJR143C';'YJR144W';'YJR145C';'YJR147↙
W';'YJR148W';'YJR150C';'YJR151C';'YJR152W';'YJR153W';'YJR155W';'YJR156C';'YJR158W';'YJR159↙
W';'YJR160C';'YJR161C';'YKL001C';'YKL002W';'YKL003C';'YKL004W';'YKL005C';'YKL006C-↙
A';'YKL006W';'YKL007W';'YKL008C';'YKL009W';'YKL010C';'YKL011C';'YKL012W';'YKL013C';'YKL014↙
C';'YKL015W';'YKL016C';'YKL017C';'YKL018W';'YKL019W';'YKL020C';'YKL021C';'YKL022C';'YKL024↙
C';'YKL025C';'YKL026C';'YKL027W';'YKL028W';'YKL029C';'YKL032C';'YKL033W';'YKL034W';'YKL035↙
W';'YKL037W';'YKL038W';'YKL039W';'YKL040C';'YKL041W';'YKL042W';'YKL043W';'YKL045W';'YKL046↙
C';'YKL048C';'YKL049C';'YKL050C';'YKL051W';'YKL052C';'YKL053C-↙
A';'YKL054C';'YKL055C';'YKL056C';'YKL057C';'YKL058W';'YKL059C';'YKL060C';'YKL062W';'YKL064↙
W';'YKL065C';'YKL067W';'YKL068W';'YKL069W';'YKL072W';'YKL073W';'YKL074C';'YKL078W';'YKL079↙
W';'YKL080W';'YKL081W';'YKL082C';'YKL084W';'YKL085W';'YKL086W';'YKL087C';'YKL088W';'YKL089↙
W';'YKL090W';'YKL091C';'YKL092C';'YKL093W';'YKL094W';'YKL095W';'YKL096W';'YKL096W-↙
A';'YKL098W';'YKL099C';'YKL101W';'YKL103C';'YKL104C';'YKL105C';'YKL106W';'YKL108W';'YKL109↙
W';'YKL110C';'YKL112W';'YKL113C';'YKL114C';'YKL116C';'YKL117W';'YKL119C';'YKL120W';'YKL122↙
C';'YKL124W';'YKL125W';'YKL126W';'YKL127W';'YKL128C';'YKL129C';'YKL130C';'YKL132C';'YKL134↙
C';'YKL135C';'YKL137W';'YKL138C';'YKL138C-↙
A';'YKL139W';'YKL140W';'YKL141W';'YKL142W';'YKL143W';'YKL144C';'YKL145W';'YKL146W';'YKL148↙
C';'YKL149C';'YKL150W';'YKL151C';'YKL152C';'YKL154W';'YKL155C';'YKL156W';'YKL157W';'YKL159↙
C';'YKL160W';'YKL161C';'YKL163W';'YKL164C';'YKL165C';'YKL166C';'YKL167C';'YKL168C';'YKL170↙
W';'YKL171W';'YKL172W';'YKL173W';'YKL174C';'YKL175W';'YKL176C';'YKL178C';'YKL179C';'YKL180↙
W';'YKL181W';'YKL182W';'YKL183W';'YKL184W';'YKL185W';'YKL186C';'YKL188C';'YKL189W';'YKL190↙
W';'YKL191W';'YKL192C';'YKL193C';'YKL194C';'YKL195W';'YKL196C';'YKL197C';'YKL198C';'YKL201↙
C';'YKL203C';'YKL204W';'YKL205W';'YKL206C';'YKL207W';'YKL208W';'YKL209C';'YKL210W';'YKL211↙
C';'YKL212W';'YKL213C';'YKL214C';'YKL215C';'YKL216W';'YKL217W';'YKL218C';'YKL219W';'YKL220↙
C';'YKL221W';'YKL222C';'YKL224C';'YKR001C';'YKR002W';'YKR003W';'YKR004C';'YKR006C';'YKR007↙
W';'YKR008W';'YKR009C';'YKR010C';'YKR013W';'YKR014C';'YKR016W';'YKR017C';'YKR019C';'YKR020↙
W';'YKR021W';'YKR022C';'YKR024C';'YKR025W';'YKR026C';'YKR027W';'YKR028W';'YKR029C';'YKR030↙
W';'YKR031C';'YKR034W';'YKR035W-↙
A';'YKR036C';'YKR037C';'YKR038C';'YKR039W';'YKR041W';'YKR042W';'YKR043C';'YKR044W';'YKR046↙
C';'YKR048C';'YKR049C';'YKR050W';'YKR052C';'YKR053C';'YKR054C';'YKR055W';'YKR056W';'YKR057↙
W';'YKR058W';'YKR059W';'YKR060W';'YKR061W';'YKR062W';'YKR063C';'YKR064W';'YKR065C';'YKR066↙
C';'YKR067W';'YKR068C';'YKR069W';'YKR071C';'YKR072C';'YKR074W';'YKR076W';'YKR077W';'YKR078↙
W';'YKR079C';'YKR080W';'YKR081C';'YKR082W';'YKR083C';'YKR084C';'YKR085C';'YKR086W';'YKR087↙
C';'YKR088C';'YKR089C';'YKR090W';'YKR091W';'YKR092C';'YKR093W';'YKR094C';'YKR095W';'YKR095↙
W-↙
A';'YKR096W';'YKR097W';'YKR098C';'YKR099W';'YKR100C';'YKR101W';'YKR102W';'YKR103W';'YKR104↙
W';'YKR106W';'YLL001W';'YLL002W';'YLL003W';'YLL004W';'YLL005C';'YLL006W';'YLL008W';'YLL009↙
C';'YLL010C';'YLL011W';'YLL012W';'YLL013C';'YLL014W';'YLL015W';'YLL018C';'YLL018C-↙
A';'YLL019C';'YLL021W';'YLL022C';'YLL023C';'YLL024C';'YLL025W';'YLL026W';'YLL027W';'YLL028↙
W';'YLL029W';'YLL031C';'YLL032C';'YLL033W';'YLL034C';'YLL035W';'YLL036C';'YLL038C';'YLL039↙
C';'YLL040C';'YLL041C';'YLL042C';'YLL043W';'YLL045C';'YLL046C';'YLL048C';'YLL049W';'YLL050↙
C';'YLL051C';'YLL052C';'YLL055W';'YLL057C';'YLL060C';'YLL061W';'YLL062C';'YLL063C';'YLL064↙
C';'YLR002C';'YLR003C';'YLR004C';'YLR005W';'YLR006C';'YLR007W';'YLR008C';'YLR009W';'YLR010↙
C';'YLR011W';'YLR013W';'YLR014C';'YLR015W';'YLR016C';'YLR017W';'YLR018C';'YLR019W';'YLR020↙
C';'YLR021W';'YLR022C';'YLR023C';'YLR024C';'YLR025W';'YLR026C';'YLR027C';'YLR028C';'YLR029↙
C';'YLR032W';'YLR033W';'YLR034C';'YLR035C';'YLR037C';'YLR038C';'YLR039C';'YLR043C';'YLR044↙
C';'YLR045C';'YLR047C';'YLR048W';'YLR051C';'YLR052W';'YLR054C';'YLR055C';'YLR056W';'YLR057↙
W';'YLR058C';'YLR059C';'YLR060W';'YLR061W';'YLR064W';'YLR065C';'YLR066W';'YLR067C';'YLR068↙
W';'YLR069C';'YLR070C';'YLR071C';'YLR073C';'YLR074C';'YLR075W';'YLR077W';'YLR078C';'YLR079↙
W';'YLR080W';'YLR081W';'YLR082C';'YLR083C';'YLR084C';'YLR085C';'YLR086W';'YLR087C';'YLR088↙
W';'YLR089C';'YLR090W';'YLR091W';'YLR092W';'YLR093C';'YLR094C';'YLR095C';'YLR096W';'YLR097↙
C';'YLR098C';'YLR099C';'YLR099W-↙
A';'YLR100W';'YLR102C';'YLR103C';'YLR105C';'YLR106C';'YLR107W';'YLR109W';'YLR110C';'YLR113↙
```

```
W';'YLR114C';'YLR115W';'YLR116W';'YLR117C';'YLR118C';'YLR119W';'YLR120C';'YLR121C';'YLR127↙
C';'YLR128W';'YLR129W';'YLR130C';'YLR131C';'YLR132C';'YLR133W';'YLR134W';'YLR135W';'YLR136↙
C';'YLR137W';'YLR138W';'YLR139C';'YLR141W';'YLR142W';'YLR144C';'YLR145W';'YLR146C';'YLR147↙
C';'YLR148W';'YLR150W';'YLR151C';'YLR153C';'YLR154C';'YLR154W-↙
C';'YLR155C';'YLR157C';'YLR158C';'YLR160C';'YLR162W';'YLR163C';'YLR164W';'YLR165C';'YLR166↙
C';'YLR167W';'YLR168C';'YLR170C';'YLR172C';'YLR174W';'YLR175W';'YLR176C';'YLR178C';'YLR179↙
C';'YLR180W';'YLR181C';'YLR182W';'YLR183C';'YLR185W';'YLR186W';'YLR188W';'YLR189C';'YLR190↙
W';'YLR191W';'YLR192C';'YLR193C';'YLR194C';'YLR195C';'YLR196W';'YLR197W';'YLR199C';'YLR200↙
W';'YLR201C';'YLR203C';'YLR204W';'YLR205C';'YLR206W';'YLR207W';'YLR208W';'YLR209C';'YLR210↙
W';'YLR212C';'YLR213C';'YLR214W';'YLR215C';'YLR216C';'YLR218C';'YLR219W';'YLR220W';'YLR221↙
C';'YLR222C';'YLR223C';'YLR226W';'YLR227C';'YLR228C';'YLR229C';'YLR231C';'YLR233C';'YLR234↙
W';'YLR237W';'YLR238W';'YLR239C';'YLR240W';'YLR242C';'YLR243W';'YLR244C';'YLR245C';'YLR246↙
W';'YLR247C';'YLR248W';'YLR249W';'YLR250W';'YLR251W';'YLR254C';'YLR256W';'YLR258W';'YLR259↙
C';'YLR260W';'YLR262C';'YLR262C-↙
A';'YLR263W';'YLR264W';'YLR265C';'YLR266C';'YLR268W';'YLR270W';'YLR272C';'YLR273C';'YLR274↙
W';'YLR275W';'YLR276C';'YLR277C';'YLR284C';'YLR285W';'YLR286C';'YLR287C-↙
A';'YLR288C';'YLR289W';'YLR291C';'YLR292C';'YLR293C';'YLR295C';'YLR298C';'YLR299W';'YLR300↙
W';'YLR301W';'YLR303W';'YLR304C';'YLR305C';'YLR306W';'YLR307W';'YLR308W';'YLR309C';'YLR310↙
C';'YLR312W-↙
A';'YLR313C';'YLR314C';'YLR315W';'YLR316C';'YLR318W';'YLR319C';'YLR320W';'YLR321C';'YLR323↙
C';'YLR324W';'YLR325C';'YLR327C';'YLR328W';'YLR329W';'YLR330W';'YLR332W';'YLR333C';'YLR335↙
W';'YLR336C';'YLR337C';'YLR340W';'YLR341W';'YLR342W';'YLR343W';'YLR344W';'YLR347C';'YLR348↙
C';'YLR350W';'YLR351C';'YLR353W';'YLR354C';'YLR355C';'YLR356W';'YLR357W';'YLR359W';'YLR360↙
W';'YLR361C';'YLR362W';'YLR363C';'YLR364W';'YLR367W';'YLR368W';'YLR369W';'YLR370C';'YLR371↙
W';'YLR372W';'YLR373C';'YLR375W';'YLR376C';'YLR377C';'YLR378C';'YLR380W';'YLR381W';'YLR382↙
C';'YLR383W';'YLR384C';'YLR385C';'YLR386W';'YLR387C';'YLR388W';'YLR389C';'YLR390W';'YLR390↙
W-↙
A';'YLR392C';'YLR393W';'YLR394W';'YLR395C';'YLR396C';'YLR397C';'YLR398C';'YLR399C';'YLR401↙
C';'YLR403W';'YLR404W';'YLR405W';'YLR406C';'YLR409C';'YLR410W';'YLR411W';'YLR412W';'YLR414↙
C';'YLR417W';'YLR418C';'YLR420W';'YLR421C';'YLR423C';'YLR424W';'YLR425W';'YLR427W';'YLR429↙
W';'YLR430W';'YLR431C';'YLR432W';'YLR433C';'YLR435W';'YLR436C';'YLR437C';'YLR438C-↙
A';'YLR438W';'YLR439W';'YLR440C';'YLR441C';'YLR442C';'YLR443W';'YLR445W';'YLR447C';'YLR448↙
W';'YLR449W';'YLR450W';'YLR451W';'YLR452C';'YLR453C';'YLR457C';'YLR459W';'YLR461W';'YLR466↙
W';'YLR467W';'YML001W';'YML004C';'YML005W';'YML006C';'YML007W';'YML008C';'YML009C';'YML010↙
W';'YML011C';'YML012W';'YML013W';'YML014W';'YML015C';'YML016C';'YML017W';'YML019W';'YML021↙
C';'YML022W';'YML023C';'YML024W';'YML025C';'YML026C';'YML027W';'YML028W';'YML029W';'YML030↙
W';'YML031W';'YML032C';'YML034W';'YML035C';'YML036W';'YML038C';'YML041C';'YML042W';'YML043↙
C';'YML046W';'YML047C';'YML048W';'YML049C';'YML050W';'YML051W';'YML052W';'YML054C';'YML055↙
W';'YML056C';'YML057W';'YML058W';'YML058W-↙
A';'YML059C';'YML060W';'YML061C';'YML062C';'YML063W';'YML064C';'YML065W';'YML066C';'YML067↙
C';'YML068W';'YML069W';'YML070W';'YML071C';'YML072C';'YML073C';'YML074C';'YML075C';'YML076↙
C';'YML077W';'YML078W';'YML080W';'YML081C-↙
A';'YML081W';'YML085C';'YML086C';'YML087C';'YML088W';'YML091C';'YML092C';'YML093W';'YML094↙
W';'YML095C';'YML097C';'YML098W';'YML099C';'YML100W';'YML101C';'YML102W';'YML103C';'YML104↙
C';'YML105C';'YML106W';'YML107C';'YML109W';'YML110C';'YML111W';'YML112W';'YML113W';'YML114↙
C';'YML115C';'YML116W';'YML117W';'YML118W';'YML120C';'YML121W';'YML123C';'YML124C';'YML125↙
C';'YML126C';'YML127W';'YML128C';'YML129C';'YML130C';'YML132W';'YMR001C';'YMR002W';'YMR003↙
W';'YMR004W';'YMR005W';'YMR006C';'YMR008C';'YMR009W';'YMR011W';'YMR012W';'YMR013C';'YMR014↙
W';'YMR015C';'YMR016C';'YMR017W';'YMR019W';'YMR020W';'YMR021C';'YMR022W';'YMR023C';'YMR024↙
W';'YMR025W';'YMR026C';'YMR028W';'YMR029C';'YMR030W';'YMR031C';'YMR032W';'YMR033W';'YMR035↙
W';'YMR036C';'YMR037C';'YMR038C';'YMR039C';'YMR040W';'YMR041C';'YMR042W';'YMR043W';'YMR044↙
W';'YMR047C';'YMR048W';'YMR049C';'YMR052W';'YMR053C';'YMR054W';'YMR055C';'YMR056C';'YMR058↙
W';'YMR059W';'YMR060C';'YMR061W';'YMR062C';'YMR063W';'YMR064W';'YMR065W';'YMR066W';'YMR067↙
C';'YMR068W';'YMR069W';'YMR070W';'YMR071C';'YMR072W';'YMR073C';'YMR074C';'YMR075W';'YMR076↙
C';'YMR077C';'YMR078C';'YMR079W';'YMR080C';'YMR081C';'YMR083W';'YMR086W';'YMR087W';'YMR088↙
C';'YMR089C';'YMR091C';'YMR092C';'YMR093W';'YMR094W';'YMR095C';'YMR096W';'YMR097C';'YMR098↙
C';'YMR099C';'YMR100W';'YMR101C';'YMR104C';'YMR105C';'YMR106C';'YMR107W';'YMR108W';'YMR109↙
W';'YMR110C';'YMR112C';'YMR113W';'YMR114C';'YMR115W';'YMR116C';'YMR117C';'YMR119W';'YMR120↙
C';'YMR121C';'YMR123W';'YMR125W';'YMR127C';'YMR128W';'YMR129W';'YMR131C';'YMR133W';'YMR135↙
C';'YMR136W';'YMR137C';'YMR138W';'YMR139W';'YMR140W';'YMR142C';'YMR143W';'YMR145C';'YMR146↙
```

```
C';'YMR148W';'YMR149W';'YMR150C';'YMR152W';'YMR153W';'YMR154C';'YMR156C';'YMR157C';'YMR158↙
W';'YMR159C';'YMR161W';'YMR162C';'YMR163C';'YMR164C';'YMR165C';'YMR167W';'YMR168C';'YMR169↙
C';'YMR170C';'YMR171C';'YMR172W';'YMR173W';'YMR174C';'YMR175W';'YMR176W';'YMR177W';'YMR179↙
W';'YMR180C';'YMR182C';'YMR183C';'YMR184W';'YMR186W';'YMR188C';'YMR189W';'YMR190C';'YMR191↙
W';'YMR192W';'YMR193W';'YMR194C-↙
B';'YMR194W';'YMR195W';'YMR197C';'YMR198W';'YMR199W';'YMR200W';'YMR201C';'YMR202W';'YMR203↙
W';'YMR204C';'YMR205C';'YMR207C';'YMR208W';'YMR210W';'YMR211W';'YMR212C';'YMR213W';'YMR214↙
W';'YMR215W';'YMR216C';'YMR217W';'YMR218C';'YMR219W';'YMR220W';'YMR222C';'YMR223W';'YMR224↙
C';'YMR225C';'YMR226C';'YMR227C';'YMR228W';'YMR229C';'YMR230W';'YMR231W';'YMR232W';'YMR233↙
W';'YMR234W';'YMR235C';'YMR236W';'YMR237W';'YMR238W';'YMR239C';'YMR240C';'YMR241W';'YMR242↙
C';'YMR243C';'YMR244C-A';'YMR246W';'YMR247C';'YMR250W';'YMR251W';'YMR251W-↙
A';'YMR255W';'YMR256C';'YMR257C';'YMR258C';'YMR259C';'YMR260C';'YMR261C';'YMR263W';'YMR264↙
W';'YMR266W';'YMR267W';'YMR268C';'YMR269W';'YMR270C';'YMR271C';'YMR272C';'YMR273C';'YMR274↙
C';'YMR275C';'YMR276W';'YMR277W';'YMR278W';'YMR279C';'YMR280C';'YMR281W';'YMR282C';'YMR283↙
C';'YMR284W';'YMR285C';'YMR286W';'YMR287C';'YMR288W';'YMR289W';'YMR290C';'YMR291W';'YMR292↙
W';'YMR293C';'YMR294W';'YMR295C';'YMR296C';'YMR297W';'YMR298W';'YMR299C';'YMR300C';'YMR301↙
C';'YMR302C';'YMR303C';'YMR304W';'YMR305C';'YMR306W';'YMR307W';'YMR308C';'YMR309C';'YMR311↙
C';'YMR312W';'YMR313C';'YMR314W';'YMR315W';'YMR316W';'YMR318C';'YMR319C';'YMR323W';'YMR325↙
W';'YNL001W';'YNL002C';'YNL003C';'YNL004W';'YNL005C';'YNL006W';'YNL007C';'YNL008C';'YNL009↙
W';'YNL012W';'YNL014W';'YNL015W';'YNL016W';'YNL020C';'YNL021W';'YNL023C';'YNL024C-↙
A';'YNL025C';'YNL026W';'YNL027W';'YNL029C';'YNL030W';'YNL031C';'YNL032W';'YNL036W';'YNL037↙
C';'YNL038W';'YNL039W';'YNL041C';'YNL042W';'YNL044W';'YNL045W';'YNL047C';'YNL048W';'YNL049↙
C';'YNL051W';'YNL052W';'YNL053W';'YNL054W';'YNL055C';'YNL056W';'YNL059C';'YNL061W';'YNL062↙
C';'YNL063W';'YNL064C';'YNL065W';'YNL066W';'YNL067W';'YNL068C';'YNL069C';'YNL070W';'YNL071↙
W';'YNL072W';'YNL073W';'YNL074C';'YNL075W';'YNL076W';'YNL077W';'YNL078W';'YNL079C';'YNL080↙
C';'YNL081C';'YNL082W';'YNL083W';'YNL084C';'YNL085W';'YNL087W';'YNL088W';'YNL090W';'YNL091↙
W';'YNL093W';'YNL094W';'YNL096C';'YNL097C';'YNL098C';'YNL099C';'YNL100W';'YNL101W';'YNL102↙
W';'YNL103W';'YNL104C';'YNL106C';'YNL107W';'YNL110C';'YNL111C';'YNL112W';'YNL113W';'YNL116↙
W';'YNL117W';'YNL118C';'YNL119W';'YNL121C';'YNL123W';'YNL124W';'YNL125C';'YNL126W';'YNL127↙
W';'YNL128W';'YNL129W';'YNL130C';'YNL131W';'YNL132W';'YNL133C';'YNL135C';'YNL136W';'YNL137↙
C';'YNL138W';'YNL138W-↙
A';'YNL139C';'YNL141W';'YNL142W';'YNL145W';'YNL147W';'YNL148C';'YNL149C';'YNL151C';'YNL152↙
W';'YNL153C';'YNL154C';'YNL156C';'YNL157W';'YNL158W';'YNL159C';'YNL160W';'YNL161W';'YNL162↙
W';'YNL163C';'YNL164C';'YNL166C';'YNL167C';'YNL169C';'YNL172W';'YNL173C';'YNL175C';'YNL177↙
C';'YNL178W';'YNL180C';'YNL182C';'YNL183C';'YNL185C';'YNL186W';'YNL187W';'YNL188W';'YNL189↙
W';'YNL191W';'YNL192W';'YNL194C';'YNL197C';'YNL199C';'YNL200C';'YNL201C';'YNL202W';'YNL204↙
C';'YNL206C';'YNL207W';'YNL208W';'YNL209W';'YNL210W';'YNL212W';'YNL213C';'YNL214W';'YNL215↙
W';'YNL216W';'YNL218W';'YNL219C';'YNL220W';'YNL221C';'YNL222W';'YNL223W';'YNL224C';'YNL225↙
C';'YNL227C';'YNL229C';'YNL230C';'YNL231C';'YNL232W';'YNL233W';'YNL234W';'YNL236W';'YNL237↙
W';'YNL238W';'YNL239W';'YNL240C';'YNL241C';'YNL242W';'YNL243W';'YNL244C';'YNL245C';'YNL246↙
W';'YNL247W';'YNL248C';'YNL249C';'YNL250W';'YNL251C';'YNL252C';'YNL253W';'YNL254C';'YNL255↙
C';'YNL256W';'YNL257C';'YNL258C';'YNL259C';'YNL260C';'YNL261W';'YNL262W';'YNL263C';'YNL264↙
C';'YNL265C';'YNL267W';'YNL268W';'YNL269W';'YNL270C';'YNL271C';'YNL272C';'YNL273W';'YNL274↙
C';'YNL275W';'YNL277W';'YNL278W';'YNL279W';'YNL280C';'YNL281W';'YNL282W';'YNL283C';'YNL284↙
C';'YNL286W';'YNL287W';'YNL288W';'YNL289W';'YNL290W';'YNL291C';'YNL292W';'YNL293W';'YNL294↙
C';'YNL297C';'YNL298W';'YNL299W';'YNL301C';'YNL302C';'YNL304W';'YNL305C';'YNL306W';'YNL307↙
C';'YNL308C';'YNL309W';'YNL310C';'YNL311C';'YNL312W';'YNL313C';'YNL314W';'YNL315C';'YNL316↙
C';'YNL317W';'YNL318C';'YNL321W';'YNL322C';'YNL323W';'YNL325C';'YNL326C';'YNL327W';'YNL328↙
C';'YNL329C';'YNL330C';'YNL331C';'YNL332W';'YNL333W';'YNL334C';'YNL336W';'YNL339C';'YNR001↙
C';'YNR002C';'YNR003C';'YNR006W';'YNR007C';'YNR008W';'YNR009W';'YNR010W';'YNR011C';'YNR012↙
W';'YNR013C';'YNR015W';'YNR016C';'YNR017W';'YNR018W';'YNR019W';'YNR020C';'YNR022C';'YNR023↙
W';'YNR024W';'YNR026C';'YNR027W';'YNR028W';'YNR030W';'YNR031C';'YNR032C-↙
A';'YNR032W';'YNR033W';'YNR034W';'YNR035C';'YNR036C';'YNR037C';'YNR038W';'YNR039C';'YNR041↙
C';'YNR043W';'YNR044W';'YNR045W';'YNR046W';'YNR047W';'YNR048W';'YNR049C';'YNR050C';'YNR051↙
C';'YNR052C';'YNR053C';'YNR054C';'YNR055C';'YNR056C';'YNR057C';'YNR058W';'YNR059W';'YNR060↙
W';'YNR064C';'YNR067C';'YNR069C';'YNR072W';'YNR074C';'YNR075W';'YNR076W';'YOL001W';'YOL002↙
C';'YOL003C';'YOL004W';'YOL005C';'YOL006C';'YOL007C';'YOL008W';'YOL009C';'YOL010W';'YOL011↙
W';'YOL012C';'YOL013C';'YOL015W';'YOL016C';'YOL017W';'YOL018C';'YOL020W';'YOL021C';'YOL022↙
C';'YOL023W';'YOL025W';'YOL026C';'YOL027C';'YOL028C';'YOL030W';'YOL031C';'YOL032W';'YOL033↙
W';'YOL034W';'YOL038W';'YOL039W';'YOL040C';'YOL041C';'YOL042W';'YOL043C';'YOL044W';'YOL045↙
```

```
W';'YOL049W';'YOL051W';'YOL052C';'YOL052C-↙
A';'YOL053W';'YOL054W';'YOL055C';'YOL056W';'YOL057W';'YOL058W';'YOL059W';'YOL060C';'YOL061↙
W';'YOL062C';'YOL063C';'YOL064C';'YOL065C';'YOL066C';'YOL067C';'YOL068C';'YOL069W';'YOL070↙
C';'YOL071W';'YOL072W';'YOL076W';'YOL077C';'YOL077W-↙
A';'YOL078W';'YOL080C';'YOL081W';'YOL082W';'YOL083W';'YOL084W';'YOL086C';'YOL086W-↙
A';'YOL087C';'YOL088C';'YOL089C';'YOL090W';'YOL091W';'YOL092W';'YOL093W';'YOL094C';'YOL095↙
C';'YOL096C';'YOL097C';'YOL100W';'YOL101C';'YOL102C';'YOL103W';'YOL104C';'YOL105C';'YOL108↙
C';'YOL109W';'YOL110W';'YOL111C';'YOL112W';'YOL113W';'YOL115W';'YOL116W';'YOL117W';'YOL119↙
C';'YOL120C';'YOL121C';'YOL122C';'YOL123W';'YOL124C';'YOL125W';'YOL126C';'YOL127W';'YOL128↙
C';'YOL129W';'YOL130W';'YOL132W';'YOL133W';'YOL135C';'YOL136C';'YOL137W';'YOL138C';'YOL139↙
C';'YOL140W';'YOL141W';'YOL142W';'YOL143C';'YOL144W';'YOL145C';'YOL146W';'YOL147C';'YOL148↙
C';'YOL149W';'YOL151W';'YOL152W';'YOL154W';'YOL155C';'YOL156W';'YOL157C';'YOL158C';'YOL159↙
C';'YOL159C-↙
A';'YOL161C';'YOL164W';'YOL165C';'YOR001W';'YOR002W';'YOR003W';'YOR004W';'YOR005C';'YOR006↙
C';'YOR007C';'YOR008C';'YOR009W';'YOR010C';'YOR011W';'YOR014W';'YOR016C';'YOR017W';'YOR018↙
W';'YOR019W';'YOR020C';'YOR021C';'YOR023C';'YOR025W';'YOR026W';'YOR027W';'YOR028C';'YOR030↙
W';'YOR031W';'YOR032C';'YOR033C';'YOR034C';'YOR035C';'YOR036W';'YOR037W';'YOR038C';'YOR039↙
W';'YOR040W';'YOR042W';'YOR043W';'YOR044W';'YOR045W';'YOR046C';'YOR047C';'YOR048C';'YOR049↙
C';'YOR051C';'YOR052C';'YOR054C';'YOR056C';'YOR057W';'YOR058C';'YOR060C';'YOR061W';'YOR063↙
W';'YOR064C';'YOR065W';'YOR066W';'YOR067C';'YOR068C';'YOR069W';'YOR070C';'YOR071C';'YOR073↙
W';'YOR074C';'YOR075W';'YOR076C';'YOR077W';'YOR078W';'YOR079C';'YOR080W';'YOR081C';'YOR083↙
W';'YOR084W';'YOR085W';'YOR086C';'YOR087W';'YOR089C';'YOR090C';'YOR091W';'YOR092W';'YOR094↙
W';'YOR095C';'YOR096W';'YOR098C';'YOR099W';'YOR100C';'YOR101W';'YOR103C';'YOR104W';'YOR106↙
W';'YOR107W';'YOR108W';'YOR109W';'YOR110W';'YOR112W';'YOR113W';'YOR115C';'YOR116C';'YOR117↙
W';'YOR118W';'YOR119C';'YOR120W';'YOR122C';'YOR123C';'YOR124C';'YOR125C';'YOR126C';'YOR127↙
W';'YOR128C';'YOR129C';'YOR130C';'YOR132W';'YOR133W';'YOR134W';'YOR136W';'YOR137C';'YOR138↙
C';'YOR140W';'YOR141C';'YOR142W';'YOR143C';'YOR144C';'YOR145C';'YOR147W';'YOR148C';'YOR149↙
C';'YOR150W';'YOR151C';'YOR153W';'YOR155C';'YOR156C';'YOR157C';'YOR158W';'YOR159C';'YOR160↙
W';'YOR161C';'YOR162C';'YOR163W';'YOR164C';'YOR165W';'YOR166C';'YOR167C';'YOR168W';'YOR171↙
C';'YOR172W';'YOR173W';'YOR174W';'YOR175C';'YOR176W';'YOR177C';'YOR178C';'YOR179C';'YOR180↙
C';'YOR181W';'YOR182C';'YOR184W';'YOR185C';'YOR187W';'YOR188W';'YOR189W';'YOR190W';'YOR191↙
W';'YOR192C';'YOR193W';'YOR194C';'YOR195W';'YOR196C';'YOR197W';'YOR198C';'YOR201C';'YOR202↙
W';'YOR204W';'YOR205C';'YOR206W';'YOR207C';'YOR208W';'YOR209C';'YOR210W';'YOR211C';'YOR212↙
W';'YOR213C';'YOR215C';'YOR216C';'YOR217W';'YOR219C';'YOR221C';'YOR222W';'YOR223W';'YOR224↙
C';'YOR226C';'YOR227W';'YOR228C';'YOR229W';'YOR230W';'YOR231W';'YOR232W';'YOR233W';'YOR234↙
C';'YOR236W';'YOR237W';'YOR239W';'YOR241W';'YOR242C';'YOR243C';'YOR244W';'YOR245C';'YOR246↙
C';'YOR247W';'YOR249C';'YOR250C';'YOR251C';'YOR252W';'YOR253W';'YOR254C';'YOR255W';'YOR256↙
C';'YOR257W';'YOR258W';'YOR259C';'YOR260W';'YOR261C';'YOR262W';'YOR264W';'YOR265W';'YOR266↙
W';'YOR267C';'YOR269W';'YOR270C';'YOR272W';'YOR273C';'YOR274W';'YOR275C';'YOR276W';'YOR278↙
W';'YOR279C';'YOR280C';'YOR281C';'YOR283W';'YOR284W';'YOR285W';'YOR286W';'YOR287C';'YOR288↙
C';'YOR290C';'YOR291W';'YOR293W';'YOR294W';'YOR295W';'YOR297C';'YOR298C-↙
A';'YOR298W';'YOR299W';'YOR301W';'YOR302W';'YOR303W';'YOR304W';'YOR305W';'YOR306C';'YOR307↙
C';'YOR308C';'YOR310C';'YOR311C';'YOR312C';'YOR313C';'YOR315W';'YOR316C';'YOR317W';'YOR319↙
W';'YOR320C';'YOR321W';'YOR322C';'YOR323C';'YOR324C';'YOR326W';'YOR327C';'YOR328W';'YOR329↙
C';'YOR330C';'YOR332W';'YOR334W';'YOR335C';'YOR336W';'YOR337W';'YOR339C';'YOR340C';'YOR341↙
W';'YOR344C';'YOR346W';'YOR347C';'YOR348C';'YOR349W';'YOR350C';'YOR351C';'YOR352W';'YOR353↙
C';'YOR354C';'YOR355W';'YOR356W';'YOR357C';'YOR358W';'YOR359W';'YOR360C';'YOR361C';'YOR362↙
C';'YOR363C';'YOR367W';'YOR368W';'YOR369C';'YOR370C';'YOR371C';'YOR372C';'YOR373W';'YOR374↙
W';'YOR375C';'YOR377W';'YOR380W';'YOR381W';'YOR382W';'YOR383C';'YOR384W';'YOR386W';'YOR388↙
C';'YOR391C';'YOR393W';'YOR394W';'YPL001W';'YPL002C';'YPL003W';'YPL004C';'YPL005W';'YPL006↙
W';'YPL007C';'YPL008W';'YPL009C';'YPL010W';'YPL011C';'YPL012W';'YPL013C';'YPL015C';'YPL016↙
W';'YPL017C';'YPL018W';'YPL019C';'YPL020C';'YPL021W';'YPL022W';'YPL023C';'YPL024W';'YPL026↙
C';'YPL027W';'YPL028W';'YPL029W';'YPL030W';'YPL031C';'YPL032C';'YPL033C';'YPL036W';'YPL037↙
C';'YPL038W';'YPL040C';'YPL042C';'YPL043W';'YPL045W';'YPL046C';'YPL047W';'YPL048W';'YPL049↙
C';'YPL050C';'YPL051W';'YPL052W';'YPL053C';'YPL054W';'YPL055C';'YPL057C';'YPL058C';'YPL059↙
W';'YPL060W';'YPL061W';'YPL063W';'YPL064C';'YPL065W';'YPL066W';'YPL069C';'YPL070W';'YPL072↙
W';'YPL074W';'YPL075W';'YPL076W';'YPL078C';'YPL079W';'YPL081W';'YPL082C';'YPL083C';'YPL084↙
W';'YPL085W';'YPL086C';'YPL087W';'YPL089C';'YPL090C';'YPL091W';'YPL092W';'YPL093W';'YPL094↙
C';'YPL095C';'YPL096C-↙
A';'YPL096W';'YPL097W';'YPL098C';'YPL099C';'YPL100W';'YPL101W';'YPL103C';'YPL104W';'YPL105↙
```

```
C';'YPL106C';'YPL110C';'YPL111W';'YPL112C';'YPL115C';'YPL116W';'YPL117C';'YPL118W';'YPL119↙
C';'YPL120W';'YPL121C';'YPL122C';'YPL123C';'YPL124W';'YPL125W';'YPL126W';'YPL127C';'YPL128↙
C';'YPL129W';'YPL130W';'YPL131W';'YPL132W';'YPL133C';'YPL134C';'YPL135W';'YPL137C';'YPL138↙
C';'YPL139C';'YPL140C';'YPL141C';'YPL143W';'YPL144W';'YPL145C';'YPL146C';'YPL147W';'YPL148↙
C';'YPL149W';'YPL151C';'YPL152W';'YPL153C';'YPL154C';'YPL155C';'YPL156C';'YPL157W';'YPL158↙
C';'YPL159C';'YPL160W';'YPL161C';'YPL163C';'YPL164C';'YPL165C';'YPL166W';'YPL167C';'YPL169↙
C';'YPL170W';'YPL171C';'YPL172C';'YPL173W';'YPL174C';'YPL175W';'YPL176C';'YPL177C';'YPL178↙
W';'YPL179W';'YPL180W';'YPL181W';'YPL183C';'YPL183W-↙
A';'YPL184C';'YPL186C';'YPL187W';'YPL188W';'YPL189C-↙
A';'YPL189W';'YPL190C';'YPL192C';'YPL193W';'YPL194W';'YPL195W';'YPL196W';'YPL198W';'YPL200↙
W';'YPL201C';'YPL202C';'YPL203W';'YPL204W';'YPL206C';'YPL207W';'YPL208W';'YPL209C';'YPL210↙
C';'YPL211W';'YPL212C';'YPL213W';'YPL214C';'YPL215W';'YPL217C';'YPL218W';'YPL219W';'YPL220↙
W';'YPL221W';'YPL223C';'YPL224C';'YPL225W';'YPL226W';'YPL227C';'YPL228W';'YPL230W';'YPL231↙
W';'YPL232W';'YPL233W';'YPL234C';'YPL235W';'YPL236C';'YPL237W';'YPL239W';'YPL240C';'YPL241↙
C';'YPL242C';'YPL243W';'YPL244C';'YPL246C';'YPL248C';'YPL249C';'YPL249C-↙
A';'YPL250C';'YPL252C';'YPL253C';'YPL254W';'YPL255W';'YPL256C';'YPL258C';'YPL259C';'YPL262↙
W';'YPL263C';'YPL265W';'YPL266W';'YPL267W';'YPL268W';'YPL269W';'YPL270W';'YPL271W';'YPL273↙
W';'YPL274W';'YPL281C';'YPL282C';'YPL283C';'YPR001W';'YPR002W';'YPR004C';'YPR005C';'YPR006↙
C';'YPR007C';'YPR008W';'YPR009W';'YPR010C';'YPR016C';'YPR017C';'YPR018W';'YPR019W';'YPR020↙
W';'YPR021C';'YPR023C';'YPR024W';'YPR025C';'YPR026W';'YPR028W';'YPR029C';'YPR030W';'YPR031↙
W';'YPR032W';'YPR033C';'YPR034W';'YPR035W';'YPR036W';'YPR036W-↙
A';'YPR037C';'YPR040W';'YPR041W';'YPR042C';'YPR043W';'YPR045C';'YPR046W';'YPR047W';'YPR048↙
W';'YPR049C';'YPR051W';'YPR052C';'YPR054W';'YPR055W';'YPR056W';'YPR057W';'YPR058W';'YPR060↙
C';'YPR061C';'YPR062W';'YPR065W';'YPR066W';'YPR067W';'YPR068C';'YPR069C';'YPR070W';'YPR072↙
W';'YPR073C';'YPR074C';'YPR075C';'YPR079W';'YPR080W';'YPR081C';'YPR082C';'YPR083W';'YPR085↙
C';'YPR086W';'YPR088C';'YPR091C';'YPR093C';'YPR094W';'YPR095C';'YPR096C';'YPR097W';'YPR098↙
C';'YPR100W';'YPR101W';'YPR102C';'YPR103W';'YPR104C';'YPR105C';'YPR106W';'YPR107C';'YPR108↙
W';'YPR110C';'YPR111W';'YPR112C';'YPR113W';'YPR115W';'YPR116W';'YPR118W';'YPR119W';'YPR120↙
C';'YPR121W';'YPR122W';'YPR124W';'YPR125W';'YPR127W';'YPR128C';'YPR129W';'YPR131C';'YPR132↙
W';'YPR133C';'YPR133W-↙
A';'YPR134W';'YPR135W';'YPR137W';'YPR138C';'YPR139C';'YPR140W';'YPR141C';'YPR143W';'YPR144↙
C';'YPR145W';'YPR148C';'YPR149W';'YPR151C';'YPR152C';'YPR153W';'YPR154W';'YPR155C';'YPR156↙
C';'YPR158W';'YPR159W';'YPR160W';'YPR161C';'YPR162C';'YPR163C';'YPR164W';'YPR165W';'YPR166↙
C';'YPR167C';'YPR168W';'YPR169W';'YPR171W';'YPR173C';'YPR175W';'YPR176C';'YPR178W';'YPR179↙
C';'YPR180W';'YPR181C';'YPR182W';'YPR183W';'YPR184W';'YPR185W';'YPR186C';'YPR187W';'YPR188↙
C';'YPR189W';'YPR190C';'YPR191W';'YPR192W';'YPR193C';'YPR194C';'YPR198W';'YPR199C';'YPR200↙
C';'YPR201W';'YPR204W';};

end
```
